# Tuneable quantum spin Hall states in confined 1*T'* transition metal dichalcogenides

**DOI:** 10.1038/s41598-020-63450-5

**Published:** 2020-04-21

**Authors:** Biswapriyo Das, Diptiman Sen, Santanu Mahapatra

**Affiliations:** 1 Nano-Scale Device Research Laboratory, Department of Electronic Systems Engineering, Indian Institute of Science (IISc) Bangalore, Bangalore, 560012 India; 2 Centre for High Energy Physics, Indian Institute of Science (IISc) Bangalore, Bangalore, 560012 India

**Keywords:** Applied physics, Condensed-matter physics, Quantum physics, Theoretical physics

## Abstract

Investigation of quantum spin Hall states in 1*T'* phase of the monolayer transition metal dichalcogenides has recently attracted the attention for its potential in nanoelectronic applications. While most of the theoretical findings in this regard deal with infinitely periodic crystal structures, here we employ density functional theory calculations and $k.p$ Hamiltonian based continuum model to unveil the bandgap opening in the edge-state spectrum of finite width molybdenum disulphide, molybdenum diselenide, tungsten disulphide and tungsten diselenide. We show that the application of a perpendicular electric field simultaneously modulates the band gaps of bulk and edge-states. We further observe that tungsten diselenide undergoes a semi-metallic intermediate state during the phase transition from topological to normal insulator. The tuneable edge conductance, as obtained from the Landauer-Büttiker formalism, exhibits a monotonous increasing trend with applied electric field for deca-nanometer molybdenum disulphide, whereas the trend is opposite for other cases.

## Introduction

Topological insulators (TI)^1–4^ have emerged as a relatively new quantum state of matter, characterized by gapped (insulating) bulk states and gapless (highly conducting) edge/surface states according to the bulk-boundary correspondence. The ‘topological’ attribute in this context is the nontrivial topology of the bulk bands spanned by their characteristic electron wavefunctions. Since this nontrivial topology is a characteristic of the gapped energy states, in order to flip the topology across the interface, either the energy gap has to be closed or the symmetry property protecting the edge/surface states has to be broken. Appearance of gapless edge (2D) or surface (3D) states at the interface of a TI and a normal insulator (NI) or vacuum is thus the most fundamental property of a topologically nontrivial phase.

Quantum spin Hall insulators (QSHI) or 2D TIs, as originally proposed by Kane and Mele^5,6^, features spin-polarized ‘helical’ edge states with opposite momentum on each side of the sample to form a Kramer’s pair. According to Kane and Mele^5^, graphene was predicted to be a QSHI with a finite gap opening at Dirac point due to spin-orbit coupling (SOC). Unfortunately, very weak SOC in graphene rendered it impossible to experimentally verify their prediction. However, in 2006, the existence of quantum spin Hall (QSH) state was theoretically predicted by Bernevig, Hughes and Zhang (BHZ)^7^ and later experimentally demonstrated by König *et al*.^8^ for HgTe/CdTe quantum wells. Thenceforth several other 2D TI materials have been reported such as InAs/GaSb^9,10^ quantum wells, bismuthene^11,12^, functionalized Bi/Sb films^13^, monolayer BiX/SbX (X = H, F, Cl and Br)^14^, 2D bismuth arsenic (BiAs)^15^, arsenene^16^, arsenene oxide^17^, monolayer AsSb^18^, phosphorene^19^, silicon-based chalcogenide (Si_2_Te_2_)^20^, 2D transition-metal halides^21^, monolayer ZrTe_5_ and HfTe_5_^22^, Cu_2_Te and Ag_2_Te^23^, silicene^24,25^, germanene^26,27^, 2D SiGe^28^, stanene^29^, functionalized stanene^30^, tellurene^31^ etc. This apart, a recent high-throughput density functional theory (DFT) based study has reported^32^ thirteen new 2D materials that are candidates for QSHI such as AsCuLi_2_, Pt_2_HgSe_3_ etc. While experimental realization of many of these 2D materials is still in infancy, recent advancements in technology have aided the growth and fabrication of several such new and exotic materials with interesting quantum properties^33,34^. However, monolayer transition metal dichalcogenides (TMD), i.e. MX_2_ where M and X denotes metal (e.g. Mo, W etc.) and chalcogen (e.g. S, Se, Te etc.) atoms respectively, were first predicted to possess the topological properties by Qian *et al*.^35^ and later it was verified by several other investigations^36–48^. Based on first principles calculations and tight-binding Hamiltonian, they demonstrated^35^ that among several polytypic structures, 1*T'* phase of a monolayer TMD features band inversion in its bulk energy spectrum. It is caused by the formation of periodic doubling of metal chain in 1*T'* structures and large SOC of transition metals opens a band gap at the otherwise gapless Dirac points. They depicted the edge-state energy spectrum for 1*T'* MoS_2_ using surface Green’s function calculations and demonstrated that topological phase transition in these materials can be achieved as a result of external perturbations like electric field or strain. However, experimental findings on the other hand indicate that the topological properties of these materials can also be tuned by temperature^38^ and surface doping^41^. Later the Haeckelite crystal structures^49^ of monolayer TMDs were also found to possess topological characteristics. To be precise, time-reversal symmetry (TRS) protected topological phases in these monolayer 1*T'* TMDs are attributed by ${{\mathbb{Z}}}_{2}$ topological invariant, where ${{\mathbb{Z}}}_{2}=0$ indicates topologically trivial phase and ${{\mathbb{Z}}}_{2}=1$ denotes topologically ‘twisted’ state or QSH state. The nontrivial topology necessarily dictates the transport through edge states to be free of elastic back-scattering, thereby effectuating the edge conductance to be exactly equal to the conductance quantum ${e}^{2}/h$, where $e$ and $h$ denotes electronic charge and Planck’s constant respectively.

However, from an engineering viewpoint, it is highly desirable to devise a method to control the transport through edge states by external means which forms the notion of topological insulator field effect transistors (TIFET)^50^. The most straightforward solution would be rapidly introducing and removing the edge states through topological phase transition, which for 1*T'* TMDs has been demonstrated by applying vertical electric field^35,36^ and strain^35^. Even, if the TRS is broken either by external perturbation (e.g. magnetic field^44^) or due to the presence of magnetic impurities^51–53^ or through ‘spontaneous’ breaking^37^ by the presence of non-zero magnetic moment, the existence of edge states is also not guaranteed or even if they exist, the conductance may deviate from the quantized value. In this article, we investigate another possible way to modulate the edge conductance, which is by confining the material geometry. Previously, Zhou *et al*.^54^ have reported a gap opening in edge state spectrum as a result of the restriction on strip width and showed that the charge conductance gets modified as a function of the energy gap.

Here, in order to investigate the effects of quantum confinement on the edge state spectra and charge conductance of monolayer 1*T'* TMDs, we have employed a continuum model based analytical approach rather than using surface Green’s function formalism. Such analytical approach would be suitable for developing compact device models for 2D TIFETs. First, we study the topological properties of four such TMD materials, to be precise, MoS_2_, MoSe_2_, WS_2_ and WSe_2_. We obtain the bulk band structures of these materials using density functional theory (DFT) computations and thereby investigate the Rashba splitting of spin-degenerate bands as a consequence of external electric field. We then identify the critical field values for these materials which define the quantum critical points for topological phase transitions. Thereafter we calibrate a $k.p$ Hamiltonian that accurately captures the band structure subtleties of 1*T'* TMDs and represent a continuum modeling approach to obtain the edge state spectrum. We introduce the finite size effect by confining the real space geometry of the material, by virtue of which, the edge states from opposite edges couple together to introduce a gap in the edge spectrum thereby transforming the massless linear Dirac dispersions to massive Dirac hyperbolas. For confined structures, the edge conductance is found to be less than the conductance quantum and it decreases with increasing degree of confinement. It is also found that the edge conductance can be tuned as a function of external electric field only for finite width of the strips, whereas for unconfined geometry it maintains a constant value of ${e}^{2}/h$ for all electric fields.

## Results

### Crystal structure and bulk energy dispersion

**Figure 1:**
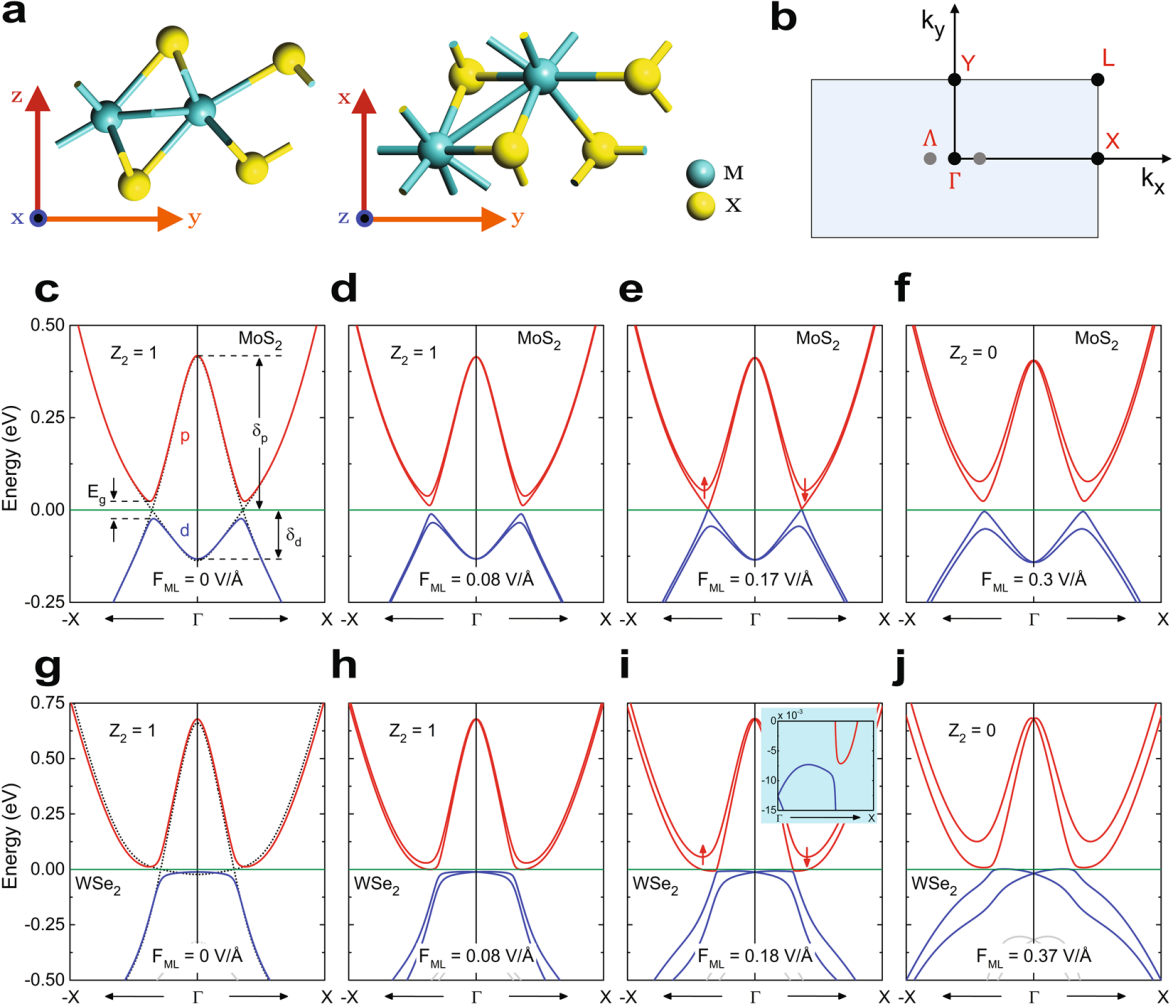
(**a**) Crystal structure of a 1*T'* MX_2_, where M and X denote metal (e.g. Mo, W etc.) and chalcogen (e.g. S, Se, Te etc.) atoms respectively. The 1*T'* structure is formed by lattice distortion of dynamically unstable 1*T* structure. (**b**) First Brillouin zone of 1*T'* MX_2_, showing the high-symmetry points Γ, X, Y and L. Γ is the zone centre where the bulk energy dispersion undergoes a band inversion. The fundamental band gap occurs at a point Λ along the ${k}_{x}$ axis. (**c**–**f**) Illustrate the bulk band structures of MoS_2_ respectively under the electric fields *F*_*ML*_ = 0, 0.08, 0.17 and 0.3 V/Å . In all of these figures red lines represent conduction bands and blue lines represent valence bands. The Fermi level, set to zero, is represented by green line. (**c**) Depicts that near Γ point the conduction band is mainly contributed by $p$ orbitals of sulphur and valence band is mainly composed of $d$ orbitals of molybdenum indicating band inversion. The effect of spin-orbit coupling is to open an energy gap near the Λ point in the otherwise gapless bulk dispersion. The black dotted line in (**c**) indicates the dispersion without considering SOC. The inverted band gap $\delta$ is the sum of contributions from conduction $({\delta }_{p})$ and valence $({\delta }_{d})$ bands. As shown in (**d**–**f**), the effect of external electric field is to introduce Rashba splitting between spin-degenerate bands thereby lifting the degeneracy. As the field strength is increased, eventually the band gap closes at ${F}_{ML}=0.17$ V/Å and in response to further increase in ${F}_{ML}$, the gap reopens with degeneracy remaining lifted. The states in (**c**) and (**d**) are topological with ${{\mathbb{Z}}}_{2}=1$ and the state in (**f**) is topologically trivial $({{\mathbb{Z}}}_{2}=0)$. However in (**e**), the topological invariant is ill-defined indicating the quantum critical point for topological phase transition. (**g**) – (**j**) demonstrate the similar characteristics of bulk energy dispersions for WSe_2_. The colour scheme for representation is same as stated before. However, it can be seen in the inset of (**i**) that as the electric field approaches the critical value, WSe_2_ tends to become an indirect semiconductor.

Monolayer TMDs are known to possess several polytypic structures e.g. 1 *H* (trigonal prismatic coordination), 1*T* (octahedral coordination) and 1*T'* (distorted 1*T* structure), among which, 1*T* phase, owing to its dynamical instability^35,55,56^, undergoes a spontaneous lattice distortion to transform into stable 1*T'* structure. Probably the most subtle feature of this 1*T'* phase is the inverted bandgap at the Brillouin zone center (Γ), that occurs as a consequence of periodic doubling of transition metal chain, lowering the metal *d* orbitals below chalcogen *p* orbitals in the energy scale. Shown in Fig. 1(a,b) are the crystal structures and rectangular first Brillouin zone (FBZ) of 1*T'* TMD. The FBZ has four time-reversal invariant momentum (TRIM) points, labeled in Fig. 1(b) as Γ, X, Y and L, while the fundamental bandgap appears at a point Λ along the ${k}_{x}$ axis. First-principles based calculations were conducted (see methods for details) for the aforesaid TMD materials in order to probe their electronic band structures. The bulk energy dispersions of 1*T'* MoS_2_ under varying perpendicular electric fields are depicted in Fig. 1(c–f) and the same for 1*T'* WSe_2_ are shown in Fig. 1(g–j), while dispersions of MoSe_2_ and WS_2_ in 1*T'* phase can be found in Supplementary Figs. 1 and 2 respectively. For all of these bulk dispersion profiles the Fermi energy is set to zero. In comparison to 1*T'* MoS_2_, the topmost valence band of WSe_2_ is ‘flatter’ owing to its heavier hole effective mass (see Table 1). Figure 1(c) depicts the band structure of monolayer 1*T'* MoS_2_ in absence of external electric field. For 1*T'* TMDs, the electronic contribution to the valence and conduction bands near Γ point mainly comes from metal *d* and chalcogen *p* orbitals respectively, indicating a band inversion^35^ at zone center with an inverted bandgap of $\delta ={\delta }_{p}+{\delta }_{d}$, where ${\delta }_{p}$ and ${\delta }_{d}$ are corresponding energies of lowest conduction and highest valence bands at Γ point with respect to zero energy level. The Dirac-like linear dispersion (represented by black dotted lines) appears at Λ point along Γ – X direction in absence of SOC and the role of high SOC of the transition metal atoms is to introduce a finite energy gap $({E}_{g})$ at Λ making the bulk system gapped. Numeric values of ${E}_{g}$, ${\delta }_{p}$ and ${\delta }_{d}$ for the four TMD materials are tabulated in Table 1, which are in good agreement with the previously reported^35^ data. The external electric field is applied along out-of-plane $z$ direction by introducing an electric potential shift between two metallic electrodes residing on opposite sides of the monolayer TMD material which is spaced out form them by sufficiently thick vacuum isolations. We denote the electric field in the vacuum region as ${F}_{VAC}$ and in the monolayer TMD as ${F}_{ML}$ and calculate these quantities following the method prescribed by Jelver *et al*.^37^. The application of vertical electric field effectuates a strong Rashba splitting of the otherwise degenerate valence and conduction bands near the fundamental band gap as illustrated in Fig. 1(d–f,h–j). As a result, the bulk band gap decreases and at a particular value of ${F}_{ML}$, called the critical electric field for topological phase transition $({F}_{C})$, the dispersion becomes gapless. The numeric values of ${F}_{C}$ for MoS_2_, MoSe_2_, WS_2_ and WSe_2_ were found to be ±0.17 V/Å, ±0.12 V/Å, ±0.35 V/Å and ±0.18 V/Å respectively. The numeric value of *F*_*C*_ = ±0.17 V/Å as obtained for MoS_2_ is slightly different form previously reported data^35^, which may originate from different simulation methodologies. However, for ${F}_{ML} > {F}_{C}$, the band gap reopens at the Λ point along Γ – X direction, but the spin degeneracy remains lifted. Inside the regime $|{F}_{ML}| < {F}_{C}$, all four of 1*T'* MX_2_ under consideration are topologically non-trivial $({{\mathbb{Z}}}_{2}=1)$ and outside that regime, i.e. for $|{F}_{ML}| > {F}_{C}$ the phase becomes trivial $({{\mathbb{Z}}}_{2}=0)$. However, exactly at ${F}_{ML}=\pm \,{F}_{C}$, the quantum phase is ill-defined because the bulk spectra in this case becomes gapless. Based on parity criteria of the valence bands, the ${{\mathbb{Z}}}_{2}$ indices for all of these materials were calculated by Qian *et al*.^35^. The existence of topological phase for $|{F}_{ML}| < {F}_{C}$ will be verified in the following section by probing the edge state spectrum. Although the electric field has similar effects on all four 1*T'* TMDs, viz. MoS_2_, MoSe_2_, WS_2_ and WSe_2_, it was found that WSe_2_ tends to become an indirect semiconductor as ${F}_{ML}$ reaches $\pm {F}_{C}$ as shown in the inset of Fig. 1(i).

**Table 1:** Material-specific parameters.

	*δ*_*p*_ (eV)	*δ*_*d*_ (eV)	*E*_*g*_ (eV)	$\frac{{{\boldsymbol{m}}}_{{\boldsymbol{x}}}^{{\boldsymbol{p}}}}{{{\boldsymbol{m}}}_{{\bf{0}}}}$	$\frac{{{\boldsymbol{m}}}_{{\boldsymbol{y}}}^{{\boldsymbol{p}}}}{{{\boldsymbol{m}}}_{{\bf{0}}}}$	$\frac{{{\boldsymbol{m}}}_{{\boldsymbol{x}}}^{{\boldsymbol{d}}}}{{{\boldsymbol{m}}}_{{\bf{0}}}}$	$\frac{{{\boldsymbol{m}}}_{{\boldsymbol{y}}}^{{\boldsymbol{d}}}}{{{\boldsymbol{m}}}_{{\bf{0}}}}$	*v*_1_ (×10^5^ m/s)	*v*_2_ (×10^5^ m/s)	*α* (eÅ)
MoS_2_	−0.417	−0.132	0.047	0.29	0.48	0.92	2.32	0.230	3.383	0.159
MoSe_2_	−0.719	−0.041	0.030	0.17	0.28	3.14	2.65	0.285	3.421	0.272
WS_2_	−0.145	−0.023	0.046	0.28	0.53	8.20	3.20	0.845	2.931	0.173
WSe_2_	−0.678	−0.012	0.023	0.16	0.36	8.40	3.28	0.380	3.542	0.241

*m*_0_ is the rest mass of electron.

### Sample geometry and topological phase transition

**Figure 2:**
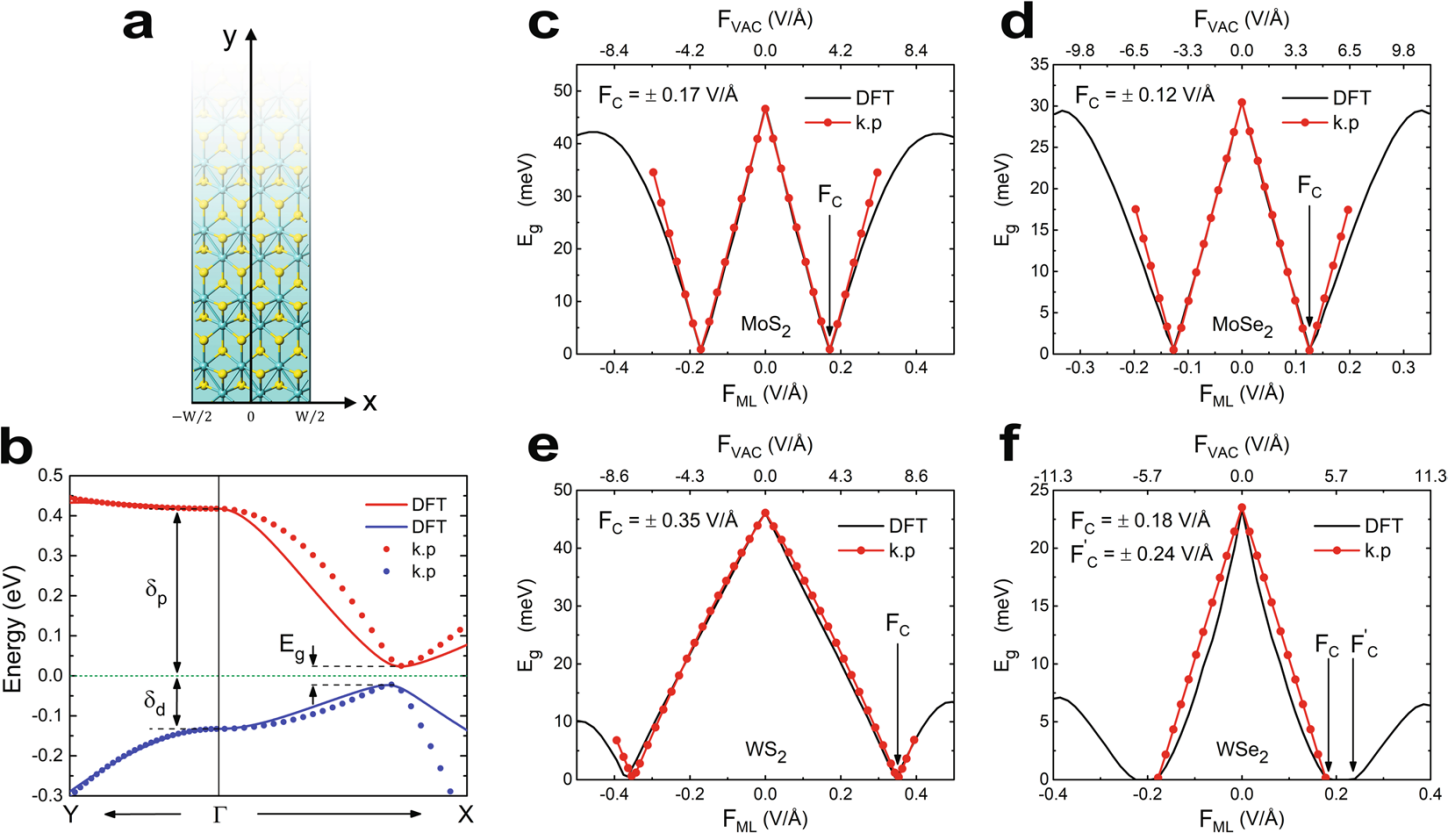
(**a**) Sample geometry under study, where the width is limited to $W$ in $x$ direction whereas it is infinite in $y$ direction. (**b**) Calibration of the $k.p$ Hamiltonian against the data obtained from DFT calculations. Here the red lines represent conduction band and blue lines represent valence band, whereas, the solid and dotted lines respectively indicate the results obtained from DFT calculations and data obtained after calibrating the $k.p$ Hamiltonian. The Fermi level, set to zero, is represented by green dashed line. (**c**–**f**) Demonstrate the topological phase transition of MoS_2_, MoSe_2_, WS_2_ and WSe_2_ respectively as a result of applied electric field. In (**c**–**f**), black solid lines represent the DFT data and red lines with data markers represent the $k.p$ Hamiltonian. The critical electric fields ${F}_{C}$ were found to be $\pm \,0.17$ V/Å, $\pm \,0.12$ V/Å, ±0.35 V/Å and ±0.18 V/Å respectively for MoS_2_, MoSe_2_, WS_2_ and WSe_2_. (c) – (f) also showcase the calibration of bulk band gaps of respective materials obtained from analytical formulation with the corresponding DFT data. The material specific electrical parameter $\alpha$ was obtained from these calibrations. However, as shown in (f), WSe_2_ acquires an intermediate semi-metallic phase for ${F}_{C}\le |{F}_{ML}|\le {F{\prime} }_{C}$ during the transition from topological to non-topological state. The concerned $k.p$ Hamiltonian fails to describe this intermediate state.

In order to investigate the effects of real-space confinement on topological phases, we consider the sample geometry as illustrated in Fig. 2(a), where the sample length is infinite along $y$ direction, but width of the material has been limited to $W$ in $x$ direction. It is to be noted that by the term ‘edge’, throughout the article we represent only that type of physical termination where TRS is preserved. Also the absence of any form of magnetic perturbation (e.g. introduction of magnetic impurities) in our model implies that TRS is always preserved. The spatial confinement of material geometry severely affects the edge-state dispersion by introducing an energy gap in the otherwise gapless edge spectra. For analytical calculation of edge state spectrum, a low energy $k.p$ Hamiltonian (see methods) was calibrated with the bulk dispersion, obtained from self-consistent DFT calculations as shown in Fig. 2(b) for MoS_2_. Details of the calibration procedure is provided in the methods section. Figure 2(c–f) demonstrate the topological phase transitions of all materials as well as comparison between the numeric values of their bulk bandgaps as obtained from DFT computations and analytical calculations from $k.p$ Hamiltonian. It signifies that the Hamiltonian accurately describes the low-energy spectrum of the bulk. Although the Hamiltonian works well within the electric fields ±*F*_*C*_ (which defines the quantum critical point for topological phase transition), it deviates from the actual result as ${F}_{ML}$ goes far beyond $\pm \,{F}_{C}$. However, it was observed that unlike other three TMDs under consideration, WSe_2_ acquires an intermediate semi-metallic gapless state during the transition from topological to non-topological phase. This semi-metallic phase appears in the regime ${F}_{C}\le |{F}_{ML}|\le {F{\prime} }_{C}$, where ${F{\prime} }_{C}$ is the critical electric field for semi-metallic to non-topological phase transition. However, the $k.p$ Hamiltonian under consideration assumes that the bulk band gap can be closed only at one particular electric field (either positive or negative), i.e. it indicates the presence of only two quantum critical points $\pm \,{F}_{C}$. Therefore the Hamiltonian fails to describe this intermediate phase of WSe_2_.

### Effect of confinement on topological insulators

**Figure 3:**
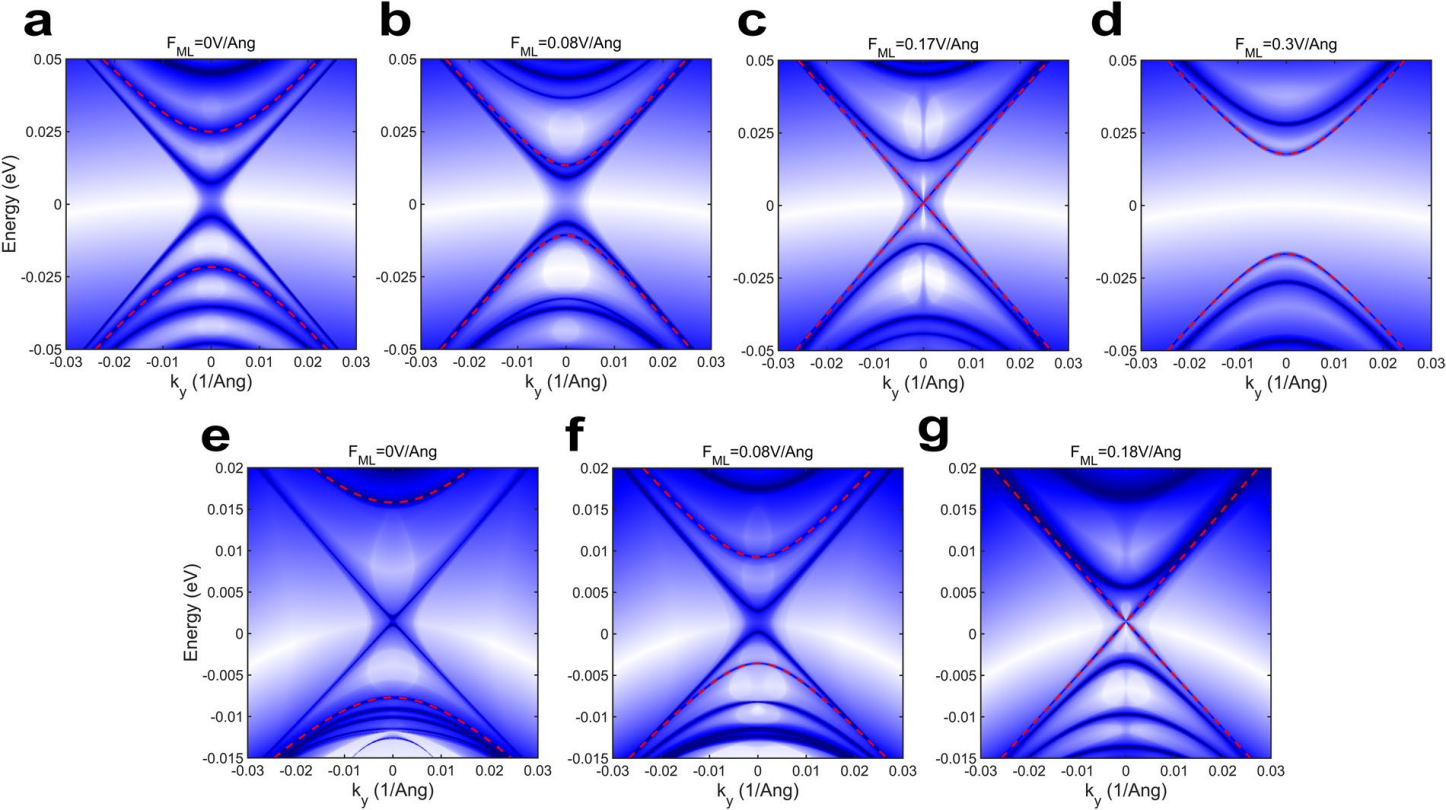
(**a–d**) are the energy spectra of edge states for 1*T'* MoS_2_ ribbon with a width of 20 nm. In all of these figures, we have plotted the functional values of coupled Eqs. (6) and (9) for given range of ${k}_{y}$ and $E$. While the color changes from white to blue to dark, the functional values change from very high to medium to zero. Therefore, the dark lines correspond to valid solutions for the energy bands. The red dashed lines correspond to bulk dispersion and the edge state dispersions can be identified as dark lines in between bulk valence and conduction bands. As shown in the figures, the effect of finite width is to introduce a gap in the otherwise gapless edge state spectrum and only when the width is small and finite, the applied electric field is able to modulate this energy gap. At ${F}_{ML}=0.17\,{\rm{V}}/\AA$, the edge states become indistinguishable from the bulk spectra indicating the phase transition point. In (**d**), ${F}_{ML}=0.3\,{\rm{V}}/\AA$, which is larger than the critical field for MoS_2_ and consequently, the phase becomes topologically trivial. Edge states vanish in this case. (**e**–**g**) Represent the edge state spectra of a WSe_2_ ribbon of width 10 nm. The colour scheme is the same as stated above. Similar arguments as stated before for MoS_2_ hold true for WSe_2_ also, except for the fact that energy spectrum for electric fields greater (or smaller) than the critical field of WSe_2_ could not be obtained since the Hamiltonian becomes invalid in that regime.

It is well known that the most subtle characteristic of a 2D TI material is the existence of chiral edge states on opposite edges of the bulk. In general, these edge states are gapless and thus their dispersions can be modeled by massless Dirac-like linear equations. On the contrary, confining the material geometry introduces a gap $({E}_{gE})$ in the otherwise gapless edge state spectrum which increases exponentially with decreasing $W$ and their massless Dirac-like linear dispersions get transformed into massive Dirac hyperbolas^54^. The edge-state energy spectra of a 1*T'* MoS_2_ ribbon with $W=20$ nm as calculated (see methods for details) from the $k.p$ Hamiltonian, are presented in Fig. 3(a–d) for increasing values of ${F}_{ML}$. Here, the solid dark areas denote valid solutions for energy eigenvalues. The red dashed lines represent the highest valence and lowest conduction bands of the bulk and the edge states can be identified as isolated dark solid lines within the bulk energy gap (although in some cases the edge states may become indistinguishable from the bulk as discussed later). As mentioned earlier, due to the finite width effect, the edge state dispersion becomes gapped for ${F}_{ML} < {F}_{C}$ and the gap appears exactly at ${k}_{y}=0$. As shown in Fig. 3(a–b), the edge state is well defined for the electric fields *F*_*ML*_ = 0 V/Å and *F*_*ML*_ = 0.08 V/Å, which are less than ${F}_{C}$. At the critical field (i.e. ${F}_{ML}={F}_{C}=0.17$ V/Å), however, the edge state spectrum becomes gapless $({E}_{gE}=0)$ and it coincides with the bulk dispersion. If ${F}_{ML}$ is further increased beyond ${F}_{C}$ (or decreased beyond $-{F}_{C}$), the edge states vanish and the phase becomes topologically trivial (i.e. NI phase), which is depicted in Fig. 3(d) where ${F}_{ML}=0.3$ V/Å. Similar dispersions of edge states under varying ${F}_{ML}$ for WSe_2_ strip ($W=10$ nm) are shown in Fig. 3(e–g). However, in this case, the energy spectrum for ${F}_{ML} > 0.18$ V/Å could not be obtained since the proposed $k.p$ Hamiltonian becomes invalid as mentioned earlier. It is further observed that edge band gap ${E}_{gE}$ gets modulated as a function of ${F}_{ML}$ and this is true only for samples with finite width because for an infinite sample $(W=\infty )$ the edge-spectrum is always gapless. Thus, the role of electric field is to introduce or remove the edge states, i.e. to effectuate topological phase transition and the effect of finite width is to make ${E}_{gE} > 0$ thereby enabling its modulation. Hence, in a nutshell, ${E}_{gE}$ can be independently controlled and modulated as a function of ${F}_{ML}$ and $W$ which is later demonstrated in detail. The edge state spectra for MoSe_2_ and WS_2_ can be found in Supplementary Figs. 3 and 4 respectively.

**Figure 4:**
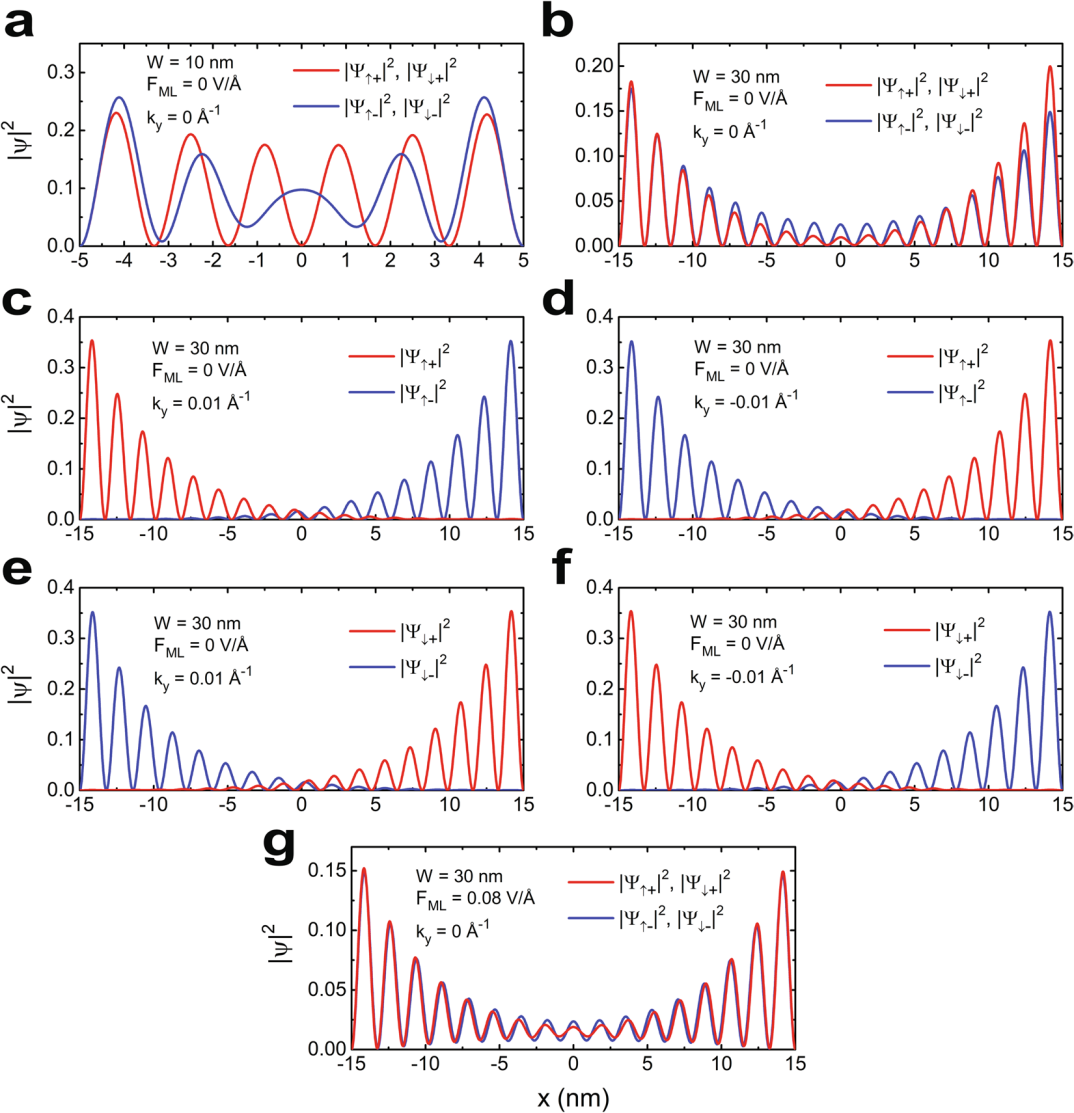
(**a**,**b**) Depict the probability density profiles of the edge state wavefunctions ${\Psi }_{\uparrow \pm }$ and ${\Psi }_{\downarrow \pm }$ for 10 nm and 30 nm wide MoS_2_ ribbons respectively. It shows that for ${k}_{y}=0$, the densities are symmetrically distributed across the space, while increased overlap between the wavefunctions for 10 nm indicates the effect of confinement. (**c**,**d**) Are respectively the density distribution profiles of spin-up states for ${k}_{y}=\pm \,0.01$ V/Å and (**e**,**f**) are the same for spin-down states with ${k}_{y}=\pm \,0.01$ V/Å. (**c**–**f**) Illustrate that the spin texture gets resolved as we move away from the centre. (**g**) Indicates that electric field does not have any significant effect on the edge state wavefunctions.

For a single edge of a TI material, the energy gap cannot be opened in the edge state spectra unless TRS is broken. However, the gap opening in our findings is a direct consequence of spatial overlap of the edge state wavefunctions ($\Psi$) from opposite sides of a finite-width sample. For infinite widths or when $W\gg {\lambda }^{-1}$ ($\lambda$ is the length scale of the wavefunction), the edge state wavefunctions are dominantly distributed near the edges of the material and corresponding probability densities (|$\Psi$|^2^) rapidly decay inside the bulk. However, when $W$ becomes comparable to or smaller than ${\lambda }^{-1}$, these wavefunctions from opposite edges can indeed overlap in space and couple together to open a gap in edge state dispersion (see methods for details). Thus for spatially confined systems with $W\le {\lambda }^{-1}$, the edge state spectra can become gapped even though TRS is preserved. For example, we find $W < 50$ nm causes significant overlap between the edge state wavefunctions of MoS_2_. To support this statement, probability densities of the normalized edge state wavefunctions of 1*T'* MoS_2_ have been plotted in Fig. 4(a,b) respectively for 10 nm and 30 nm sample widths in absence of electric field. As shown in Fig. 4(a,b), at ${k}_{y}=0$, all the density profiles are symmetrically distributed on both sides with ${|{\Psi }_{\uparrow +}|}^{2}={|{\Psi }_{\downarrow +}|}^{2}$ and ${|{\Psi }_{\uparrow -}|}^{2}={|{\Psi }_{\downarrow -}|}^{2}$ where ${\Psi }_{\uparrow +}$, ${\Psi }_{\downarrow +}$ are respectively spin-up $({\sigma }_{x}=1)$ and spin-down $({\sigma }_{x}=-1)$ electron wavefunctions for conduction band while ${\Psi }_{\uparrow -}$, ${\Psi }_{\downarrow -}$ are respectively the same for valence band with ${\sigma }_{x}$ being the first Pauli matrix. As stated earlier, the wavefunctions for finite widths do not vanish far away from the edges in the bulk, rather they overlap in space. This overlap occurs between ${\Psi }_{\uparrow \pm }(x,+{k}_{y})$ and ${\Psi }_{\uparrow \pm }(x,-{k}_{y})$, and also between similar spin-down states. As shown in Fig. 4(a), the overlap is more pronounced for $W=10$ nm than $W=30$ nm, causing larger ${E}_{gE}$ for smaller width. It is also noted that the spin texture of the edge states is not resolved at ${k}_{y}=0$. Figure 4(c–f) depicts the probability densities of the same wavefunctions at ${k}_{y}=\pm \,0.01$ Å^−1^ with $W=30$ nm and ${F}_{ML}=0$ V/Å. Quite obviously, the spin texture is resolved in this case as ${k}_{y}\ne 0$. Therefore, for any non-zero ${k}_{y}$, the states ${\Psi }_{\uparrow +}(x,+{k}_{y})$ and ${\Psi }_{\uparrow -}(x,-\,{k}_{y})$ have the same spin texture and positive velocity $({v}_{y} > 0)$ and the density distribution is dominant on one side; whereas ${\Psi }_{\uparrow +}(x,-{k}_{y})$ and ${\Psi }_{\uparrow -}(x,{k}_{y})$ have same spin texture and negative velocity $({v}_{y} < 0)$ and the density distribution is dominant on other side. The spin-down states also behave in a similar fashion. Nevertheless, near ${k}_{y}=0$ the wavefunctions couple together due to spatial confinement and the densities of ${\Psi }_{\uparrow \pm }(x,{k}_{y}=0)$ and ${\Psi }_{\downarrow \pm }(x,{k}_{y}=0)$ are distributed symmetrically on both sides, which explains the opening of energy gap in edge state spectrum exactly at ${k}_{y}=0$. However, the external electric field does not have any significant impact on the edge state wavefunctions other than increasing the amplitude of ${|\Psi |}^{2}$ near $x=0$ as shown in Fig. 4(g).

**Figure 5:**
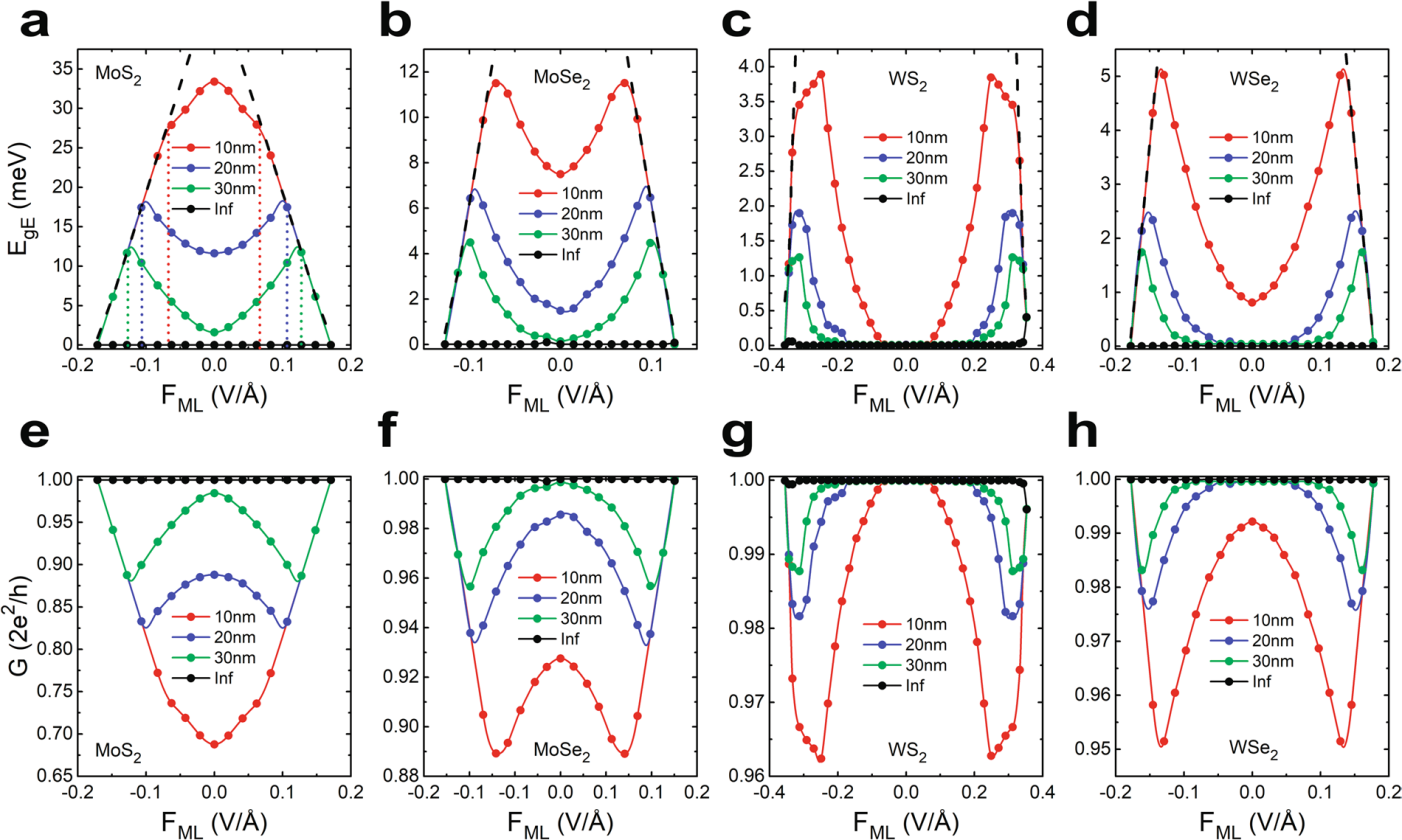
(**a**–**d**) Demonstrate the variation of edge state band gap ${E}_{gE}$ as a function of the monolayer electric field ${F}_{ML}$ for the materials MoS_2_, MoSe_2_, WS_2_ and WSe_2_ respectively. In all of these plots, the black dashed lines indicate the bulk band gap profile with respect to the field strength. The red, blue, green and black lines with data markers respectively indicate the results obtained for the sample widths 10 nm, 20 nm, 30 nm and infinite. To be noted from these figures is that for any particular field strength, ${E}_{gE}$ increases with increasing degree of confinement. Also the ‘effective critical field’ gets reduced with decreasing ribbon width. (**e**–**h**) Showcases the charge conductance profiles for the respective materials. The color scheme of representation is same as mentioned before. $G$ attains its maximum value of $2{e}^{2}/h$ for infinitely wide sample and it gets modulated by ${F}_{ML}$ only when the ribbon-width is finite.

Figure 5(a–d) represent the variation of ${E}_{gE}$ as a function of ${F}_{ML}$ $(\,-\,{F}_{C}\le {F}_{ML}\le {F}_{C})$ with $W$ as a parameter (ranging from 10 nm to infinite) respectively for the 1*T'* phases of MoS_2_, MoSe_2_, WS_2_ and WSe_2_. Here the black dashed lines indicate the variation of bulk band gap with respect to ${F}_{ML}$ of the corresponding material. It clarifies that ${E}_{gE}$ cannot be tuned at all with the electric field for an infinitely wide sample. However, except for the case of deca-nanometer MoS_2_, for a given finite width, ${E}_{gE}$ increases monotonically with increasing $|{F}_{ML}|$ until it meets the bulk band gap profile. Figure 5(a–d) also reveals that for any particular ${F}_{ML}$, ${E}_{gE}$ increases with increasing degree of confinement. Interestingly, the most peculiar characteristic is that for a given small and finite $W$, ${E}_{gE}$ coincides with ${E}_{g}$, i.e. the edge states become indistinguishable from bulk bands at an electric field much lower (higher) than ${F}_{C}$ $(-{F}_{C})$ and it becomes more prominent with decreasing values of $W$. This can be regarded as a reduction of effective ${F}_{C}$ for a small finite width, where ${F}_{C}$ actually defines the critical field for infinite geometry. These ‘effective critical fields’ for 1*T'* MoS_2_ are marked with colored dotted lines in Fig. 5(a). Thus for a TMD material with small $W$, such phase with an electric field between ‘effective critical field’ and ${F}_{C}$ becomes somewhat ambiguous in topological parlance. This apart, it is also observed that for materials with heavy electron effective mass (viz. WS_2_ and WSe_2_), the edge state spectra remain gapless at zero electric field even when the sample width is very small. For example, as shown in Fig. 5(c), ${E}_{gE}$ for WS_2_ remains zero in absence of electric field even when $W=10$ nm. Similar observations for WSe_2_ can be made from Fig. 5(d) when $W\ge 20$ nm. This is because the length scale of the wavefunctions i.e. $\lambda$ is a complex function of electron effective masses (viz. ${m}_{x}^{p}$, ${m}_{y}^{p}$, ${m}_{x}^{d}$ and ${m}_{y}^{d}$) and thus the characteristic ${\lambda }^{-1}$ becomes smaller for a material with heavier effective mass.

As the edge states deviate from their ideal linear dispersions owing to spatial confinement of strip geometry, the charge conductance $(G)$ through them should also get modified accordingly. Theoretically, the ideal charge conductance of a QSH phase is $2{e}^{2}/h$ because of the presence of two chiral 1D conducting channels at the edges of the QSH strip^8^. Following the Landauer-Büttiker formula (see methods), the numeric values of $G$ were calculated for the gapped edge states in all four materials and are presented in Fig. 5(e–h). It is noteworthy that $G$ is very sensitive to the variations of ${E}_{gE}$. As shown in these figures, $G$ attains the maximum value of $2{e}^{2}/h$ when the width is infinite, but it gradually decreases with decreasing $W$. Nevertheless, the charge conductance can only be tuned by the external field when $W$ is finite. Figure 5(e–h) demonstrate that for a given finite $W$, the conductance profile decreases (with deca-nanometer MoS_2_ being an exception) from its maximum attainable value at ${F}_{ML}=0$ V/Å with increasing $|{F}_{ML}|$ until $|{F}_{ML}|$ takes the value of ‘effective critical field’ for that width of that particular material.

## Discussion

Using first-principles based calculations and by employing a calibrated $k.p$ Hamiltonian, we have studied the effect of spatial confinement and external electric field on the QSH states in four monolayer 1*T'* TMD materials viz. MoS_2_, MoSe_2_, WS_2_ and WSe_2_. We find that the vertical electric field effectuates a topological phase transition in these materials at a particular field strength, called the critical electric field and thus it is responsible for turning the edge states ‘on’ or ‘off’. We also find that unlike the other three materials, 1*T'* WSe_2_ acquires an intermediate semi-metallic phase during the topological phase transition. However, infinite geometry of the materials renders it impossible to modulate the edge state dispersion by applying the vertical electric field. On the contrary, spatial confinement of material geometry opens a band gap in the otherwise gapless edge state spectrum, caused by the coupling between overlapping wavefunctions from opposite edges of the material. It also enables the external field to modulate the edge spectra and thereby the charge conductance. In this case, the charge conductance may deviate from its ideal value of $2{e}^{2}/h$. Finally, we conclude that the effect of confinement may prove to be a way to engineer the charge conductance through edge states, which may become useful for designing a TIFET.

## Methods

### First-principles based atomistic computations

First-principles based calculations for all four monolayer 1*T'* TMDs were carried out using the DFT code as implemented in QuantumATK^57^ in conjunction with generalized gradient approximation exchange correlation for non-collinear SOC (SOGGA) and Perdew-Burke-Ernzerhof (PBE) functional^58^. We have employed fully relativistic SG15^59,60^ (SG15-SO) norm-conserving pseudopotentials as implemented in QuantumATK database along with corresponding LCAO (linear combination of atomic orbitals) basis set of ‘medium’ accuracy for all elements. We also selected the fermion occupation method to be gaussian smearing with the electron temperatures 595 K, 1625K, 880 K and 600 K for MoS_2_, MoSe_2_, WS_2_ and WSe_2_ respectively. For Brillouin zone integration, the Monkhorst-Pack^61^ k-point samplings were set to $13\times 13\times 1$ and $11\times 11\times 1$ for MoS_2_ and MoSe_2_ respectively and $7\times 7\times 1$ for WS_2_ and WSe_2_ along with the density mesh cutoff energy being 90 Hartree for MoS_2_ and MoSe_2_, 400 Hartree for WS_2_ and 180 Hartree for WSe_2_. Maximum iteration steps for self-consistent calculations were set to 200 using Pulay mixer algorithm and we followed the fast Fourier transform (FFT) for Poisson solver. Furthermore, sufficient vacuum region of about 15 Å was provided in $z$ direction to all the structures in order to avoid spurious interactions between periodic images. The geometry optimization of unit cells of these materials including the effects of SOC were performed using LBFGS (Limited-memory Broyden-Fletcher-Goldfarb-Shanno) optimizer^62^ with maximum stress error tolerance of 0.001 eV/Å^3^ and force tolerance of 0.01 eV/Å. However, in presence of finite electric field, the geometry optimization in QuantumATK with SG15-SO pseudopotentials including the effects of SOC is a formidable task and computationally way more resource-intensive, which often develops convergence issues. Therefore the optimization of unit cells were only limited to the case of zero electric field. Nevertheless, the electric field should have negligible impact on the ionic positions as mentioned in the supplementary material of ref. ^35^. In order to investigate the effects of electric field, two metal electrodes are inserted along $z$ direction on either sides of the material with sufficient vacuum isolations, whereby an electric potential difference in these electrodes induces the electric field ${F}_{ML}$ in the monolayer TMD. Thereafter, self-consistent calculations were carried out using multi-grid Poisson solver with Dirichlet boundary condition set to the $z$ direction.

Nevertheless, despite all the atomistic calculations were carried out using PBE functional, the bulk band structures of all four 1*T'* TMDs at zero electric field, obtained using Heyd-Scuseria-Ernzerhof (HSE)^63^ hybrid functional as implemented in the VASP^64,65^ code are presented in Supplementary Fig. 5 for sake of comparison.

### Development of *k*.*p* Hamiltonian based continuum model

We start with a low-energy $k.p$ Hamiltonian $({H}_{k.p})$ for monolayer 1*T'* MX_2_ structures, as prescribed by Qian *et al*.^35^ and Liu *et al*.^36^ that follows the form:

1 $${H}_{k.p}=(\begin{array}{cccc}{E}_{p}({k}_{x},{k}_{y}) & 0 & -i{v}_{2}\hslash {k}_{y} & {v}_{1}\hslash {k}_{x}\\ 0 & {E}_{p}({k}_{x},{k}_{y}) & {v}_{1}\hslash {k}_{x} & -i{v}_{2}\hslash {k}_{y}\\ i{v}_{2}\hslash {k}_{y} & {v}_{1}\hslash {k}_{x} & {E}_{d}({k}_{x},{k}_{y}) & 0\\ {v}_{1}\hslash {k}_{x} & i{v}_{2}\hslash {k}_{y} & 0 & {E}_{d}({k}_{x},{k}_{y})\end{array})$$

where ${E}_{p}({k}_{x},{k}_{y})=-{\delta }_{p}-\frac{{\hslash }^{2}{k}_{x}^{2}}{2{m}_{x}^{p}}-\frac{{\hslash }^{2}{k}_{y}^{2}}{2{m}_{y}^{p}}$ and ${E}_{d}({k}_{x},{k}_{y})={\delta }_{d}+\frac{{\hslash }^{2}{k}_{x}^{2}}{2{m}_{x}^{d}}+\frac{{\hslash }^{2}{k}_{y}^{2}}{2{m}_{y}^{d}}$ with $\hslash$ being the modified Planck’s constant, ${m}_{x}^{p}$, ${m}_{y}^{p}$, ${m}_{x}^{d}$ and ${m}_{y}^{d}$ being corresponding effective masses in conduction $(p)$ and valence $(d)$ bands in $x$ and $y$ directions respectively and ${v}_{1},{v}_{2}$ are respectively the velocities along $x$ and $y$ directions. ${E}_{p}$ and ${E}_{d}$ basically denote the onsite energies of $p$ and $d$ orbitals respectively and the upper and lower 2×2 blocks along the off-diagonal of ${H}_{k.p}$ define the inter-band interactions between them. Numeric values of ${\delta }_{p}$ and ${\delta }_{d}$ were directly obtained from the DFT data while the effective masses and velocities were obtained by calibrating the above Hamiltonian with DFT results. All of these material-specific parameters can be found in Table 1. In our calculation $\delta (\,=\,{\delta }_{p}+{\delta }_{d}) < 0$ represents the band inversion at $\varGamma$ point.

Numerical fitting of ${H}_{k.p}$ with the data obtained from first-principles calculations was carried out for both $\Gamma \to {\rm{X}}$ and $\Gamma \to {\rm{Y}}$ directions in the FBZ using nonlinear least-squares method in conjunction with trust-region-reflective optimization algorithm^66,67^. Since precise fitting is required around both $\Gamma$ and $\Lambda$ points, it is necessary to identify a region around the $\Gamma$ point which is just sufficient to include $\Lambda$. point. Thereby, about 0.2 Å^−1^ around the $\Gamma$ point in both ${\rm{X}}$ and ${\rm{Y}}$ directions was chosen for curve-fitting. It was observed that although the Hamiltonian fits nicely in the $\Gamma \to {\rm{Y}}$ direction, it fits poorly beyond $\Lambda$ point along the $\Gamma \to {\rm{X}}$ direction, which becomes more prominent for the materials with heavy electron effectiev mass such as WS_2_ and WSe_2_.

To incorporate the effects of external electric field, i.e. the Rashba splitting of spin-degenerate bands, we consider the electric field Hamiltonian^36^ as:

2 $${H}_{F}=\alpha {F}_{ML}(\begin{array}{cccc}0 & 0 & 1 & 0\\ 0 & 0 & 0 & 1\\ 1 & 0 & 0 & 0\\ 0 & 1 & 0 & 0\end{array})$$

where ${F}_{ML}$ is the monolayer electric field induced by the potential difference between the electrodes and $\alpha$ is a material-specific electrical constant. Thus the total Hamiltonian of 1*T'* MX_2_ including the effect of electric field becomes:

3 $$H={H}_{k.p}+{H}_{F}$$

By fitting the band gap as obtained from the eigenvalues of $H$ against the band gap computed by DFT (see Fig. 2(c–f)), we get the numeric values of $\alpha$ for different materials as tabulated in Table 1.

We start the analytical derivation of edge state spectra by rewriting $H$ as:

4 $$H=({\delta }^{-}I-{\delta }^{+}{\tau }_{z})-({M}_{x}^{-}I+{M}_{x}^{+}{\tau }_{z}){k}_{x}^{2}-({M}_{y}^{-}I+{M}_{y}^{+}{\tau }_{z}){k}_{y}^{2}+{v}_{1}\hslash {k}_{x}{\tau }_{x}{\sigma }_{x}+{v}_{2}\hslash {k}_{y}{\tau }_{y}+\alpha {F}_{ML}{\tau }_{x}$$

where, ${\delta }^{\pm }=({\delta }_{d}\pm {\delta }_{p})/2$ and ${M}_{x(y)}^{\pm }=\frac{{\hslash }^{2}}{4}\left(\frac{1}{{m}_{x(y)}^{p}}\pm \frac{1}{{m}_{x(y)}^{d}}\right)$. Here $I$ stands for identity matrix, and the $4\times 4$ matrices ${\sigma }_{x}$, ${\tau }_{x}$, ${\tau }_{y}$, ${\tau }_{z}$ are mathematically represented as:

$${\sigma }_{x}=(\begin{array}{cccc}0 & 1 & 0 & 0\\ 1 & 0 & 0 & 0\\ 0 & 0 & 0 & 1\\ 0 & 0 & 1 & 0\end{array}),\,{\tau }_{x}=(\begin{array}{cccc}0 & 0 & 1 & 0\\ 0 & 0 & 0 & 1\\ 1 & 0 & 0 & 0\\ 0 & 1 & 0 & 0\end{array}),\,{\tau }_{y}=(\begin{array}{cccc}0 & 0 & -i & 0\\ 0 & 0 & 0 & -i\\ i & 0 & 0 & 0\\ 0 & i & 0 & 0\end{array}),\,{\tau }_{z}=(\begin{array}{cccc}1 & 0 & 0 & 0\\ 0 & 1 & 0 & 0\\ 0 & 0 & -1 & 0\\ 0 & 0 & 0 & -1\end{array})$$

The last term in Eq. (4) describes the electric field. For this Hamiltonian in Eq. (4), ${\sigma }_{x}$ can be found to be a good quantum number that has the eigenvalues ±1. Hence in ${\sigma }_{x}$ basis, the spin texture can be resolved and the $4\times 4$ Hamiltonian $H$ can be separated into two $2\times 2$ Hamiltonians corresponding to each eigenvalue of ${\sigma }_{x}$. We nomenclate ${\sigma }_{x}=+1$ states as ‘spin-up’ states $({\psi }_{\uparrow })$ and ${\sigma }_{x}=-1$ states as ‘spin-down’ states $({\psi }_{\downarrow })$. Now, let us consider only the spin-up Hamiltonian that reads:

5 $${H}_{\uparrow }=\left(\begin{array}{cc}-{\delta }_{p}-\frac{{\hslash }^{2}{k}_{x}^{2}}{2{m}_{x}^{p}}-\frac{{\hslash }^{2}{k}_{y}^{2}}{2{m}_{y}^{p}} & {v}_{1}\hslash {k}_{x}-i{v}_{2}\hslash {k}_{y}+\alpha {F}_{ML}\\ {v}_{1}\hslash {k}_{x}+i{v}_{2}\hslash {k}_{y}+\alpha {F}_{ML} & {\delta }_{d}+\frac{{\hslash }^{2}{k}_{x}^{2}}{2{m}_{x}^{d}}+\frac{{\hslash }^{2}{k}_{y}^{2}}{2{m}_{y}^{d}}\end{array}\right)$$

Therefore the eigenvalue problem for spin-up states becomes ${\hat{H}}_{\uparrow }{\psi }_{\uparrow }=E{\psi }_{\uparrow }$ with $E$ being the energy eigenvalue and ${\psi }_{\uparrow }$ being a two-component wavefunction $${\psi }_{\uparrow }=(\begin{array}{c}{\psi }_{1}\\ {\psi }_{2}\end{array})$$. For a strip geometry of width $W$ (see Fig. 2(a)), only ${k}_{y}$ is a good quantum number and ${k}_{x}$ should be replaced by Peierls substitution ${k}_{x}=-i\partial /\partial x$. Now, in order to obtain the energy spectrum and wavefunctions, we solve the coupled Schrödinger equations using the trial wavefunctions $${\psi }_{1,2}={e}^{\lambda x}(\begin{array}{c}1\\ 1\end{array})$$. The resulting secular equation reads

6 $$|\begin{array}{cc}\left(-{\delta }_{p}-\frac{{\hslash }^{2}{k}_{y}^{2}}{2{m}_{y}^{p}}-E\right)+\frac{{\hslash }^{2}}{2{m}_{x}^{p}}{\lambda }^{2} & -i{v}_{2}\hslash {k}_{y}+\alpha {F}_{ML}-i{v}_{1}\hslash \lambda \\ i{v}_{2}\hslash {k}_{y}+\alpha {F}_{ML}-i{v}_{1}\hslash \lambda  & \left({\delta }_{d}+\frac{{\hslash }^{2}{k}_{y}^{2}}{2{m}_{y}^{d}}-E\right)-\frac{{\hslash }^{2}}{2{m}_{x}^{d}}{\lambda }^{2}\end{array}|=0$$

It gives four roots of $\lambda$, namely ${\lambda }_{1}$, ${\lambda }_{2}$, ${\lambda }_{3}$ and ${\lambda }_{4}$. The nature of ${\lambda }_{n}$s $(n=1,2,3,4)$ determine the distribution of wavefunction in space. If ${\lambda }_{n}$s are purely imaginary, then the wavefunctions can be expressed in terms of sine and cosine functions, i.e. they span throughout the whole space indicating solutions for bulk states. But when ${\lambda }_{n}$s become real quantities, the wavefunctions are mainly distributed near the edges and they rapidly decay inside the bulk, pointing out the existence of edge states. However, in our calculation, ${\lambda }_{n}$s were found to be complex numbers with non-zero real part and therefore the resulting wavefunctions become oscillatory as well as exponentially decaying away from the edges (see Fig. 4). In general, the secular equation (Eq. (6)) turns out to be a depressed quartic equation in presence of external electric field. Nevertheless, at ${F}_{ML}=0$, it transforms into a simple quadratic equation. Next, we figure out the eigenfunctions ${\psi }_{1}$ and ${\psi }_{2}$ as functions of ${\lambda }_{n}$ as:

7 $$\begin{array}{rcl}{\psi }_{1}({\lambda }_{n}) & = & 1\\ {\psi }_{2}({\lambda }_{n}) & = & \frac{\left(-{\delta }_{p}-\frac{{\hslash }^{2}{k}_{y}^{2}}{2{m}_{y}^{p}}-E\right)+\frac{{\hslash }^{2}}{2{m}_{x}^{p}}{\lambda }_{n}^{2}}{i{v}_{2}\hslash {k}_{y}-\alpha {F}_{ML}+i{v}_{1}\hslash {\lambda }_{n}}\end{array}$$

Taking their linear combination, therefore we may construct the wavefunction as:

8 $${\psi }_{\uparrow }=\mathop{\sum }\limits_{n=1}^{4}{C}_{n}\exp ({\lambda }_{n}x)(\begin{array}{c}{\psi }_{1}({\lambda }_{n})\\ {\psi }_{2}({\lambda }_{n})\end{array})$$

where ${C}_{n}$s $(n=1,2,3,4)$ are the coefficients for linear combination. Now, applying the open boundary condition ${\psi }_{\uparrow }(x=\pm W/2,{k}_{y})=0$, we get another secular equation from the condition of having a nontrivial solution of these coefficients, that takes the following form.

9 $$\mathop{\sum }\limits_{j=1}^{3}{\varTheta }_{j}({\lambda }_{1},{\lambda }_{2},{\lambda }_{3},{\lambda }_{4})=0$$

The expressions for $${\varTheta }_{j}$$$$(j=1,2,3)$$ are given as:

10 $$\begin{array}{rcl}{\Theta }_{1} & = & \sinh \left[\frac{W}{2}({\lambda }_{1}-{\lambda }_{2})\right]\times \,\sinh \left[\frac{W}{2}({\lambda }_{3}-{\lambda }_{4})\right]\times \{{\psi }_{2}({\lambda }_{1}){\psi }_{2}({\lambda }_{2})+{\psi }_{2}({\lambda }_{3}){\psi }_{2}({\lambda }_{4})\}\\ {\Theta }_{2} & = & -\,\sinh \left[\frac{W}{2}({\lambda }_{1}-{\lambda }_{3})\right]\times \,\sinh \left[\frac{W}{2}({\lambda }_{2}-{\lambda }_{4})\right]\times \{{\psi }_{2}({\lambda }_{1}){\psi }_{2}({\lambda }_{3})+{\psi }_{2}({\lambda }_{2}){\psi }_{2}({\lambda }_{4})\}\\ {\Theta }_{3} & = & \sinh \left[\frac{W}{2}({\lambda }_{2}-{\lambda }_{3})\right]\times \,\sinh \left[\frac{W}{2}({\lambda }_{1}-{\lambda }_{4})\right]\times \{{\psi }_{2}({\lambda }_{2}){\psi }_{2}({\lambda }_{3})+{\psi }_{2}({\lambda }_{1}){\psi }_{2}({\lambda }_{4})\}\end{array}$$

Finally, the energy spectrum of the edge states can be obtained by numerically solving Eqs. (6) and (9) (see Fig. 3). The MATLAB code used to produce the dispersions in Fig. 3 is given in Supplementary Data. The same formalism can be applied to get the solutions for spin-down $({\sigma }_{x}=-1)$ states. However, as it suggests, the dispersions for spin-up and spin-down states were found to be degenerate.

On the other hand, to obtain the edge-state wavefunctions, we need the numeric values of the coefficients ${C}_{n}$. If we choose ${C}_{1}$ to be unity, then from the condition of having nontrivial solutions of the other three coefficients we can get the analytical expressions for ${C}_{2}$, ${C}_{3}$ and ${C}_{4}$ as noted below.

11 $$\begin{array}{c}{C}_{1}=1\\ {C}_{2}=-\frac{\beta \exp \left[\frac{W}{2}({\lambda }_{4}-{\lambda }_{2})\right]+\beta {\prime} \exp \left[\frac{W}{2}({\lambda }_{3}-{\lambda }_{2})\right]+\exp \left[\frac{W}{2}({\lambda }_{1}-{\lambda }_{2})\right]}{1+\gamma \exp \left[\frac{W}{2}({\lambda }_{4}-{\lambda }_{2})\right]+\gamma {\prime} \exp \left[\frac{W}{2}({\lambda }_{3}-{\lambda }_{2})\right]}\\ {C}_{3}=\beta {\prime} +\gamma {\prime} {C}_{2}\\ {C}_{4}=\beta +\gamma {C}_{2}\end{array}$$

where, *β*, *β*′, *γ* and *γ*′ are expressed as:

12 $$\begin{array}{ccc}\beta =\frac{{\psi }_{2}({\lambda }_{1})\sinh \left[\frac{W}{2}({\lambda }_{3}-{\lambda }_{1})\right]}{{\psi }_{2}({\lambda }_{4})\sinh \left[\frac{W}{2}({\lambda }_{4}-{\lambda }_{3})\right]} & , & \beta {\prime} =\frac{{\psi }_{2}({\lambda }_{1})\sinh \left[\frac{W}{2}({\lambda }_{4}-{\lambda }_{1})\right]}{{\psi }_{2}({\lambda }_{3})\sinh \left[\frac{W}{2}({\lambda }_{3}-{\lambda }_{4})\right]}\\ \gamma =\frac{{\psi }_{2}({\lambda }_{2})\sinh \left[\frac{W}{2}({\lambda }_{3}-{\lambda }_{2})\right]}{{\psi }_{2}({\lambda }_{4})\sinh \left[\frac{W}{2}({\lambda }_{4}-{\lambda }_{3})\right]} & , & \gamma {\prime} =\frac{{\psi }_{2}({\lambda }_{2})\sinh \left[\frac{W}{2}({\lambda }_{4}-{\lambda }_{2})\right]}{{\psi }_{2}({\lambda }_{3})\sinh \left[\frac{W}{2}({\lambda }_{3}-{\lambda }_{4})\right]}\end{array}$$

Finally, the normalized wavefunctions for ${\sigma }_{x}=+1$ can be written as:

13 $${\varPsi }_{\uparrow }(x,{k}_{y})=N{e}^{i{k}_{y}y}\mathop{\sum }\limits_{n=1}^{4}{C}_{n}\exp ({\lambda }_{n}x)(\begin{array}{c}{\psi }_{1}({\lambda }_{n})\\ {\psi }_{2}({\lambda }_{n})\end{array})$$

where $N$ is the normalization constant. Again, using the same formalism as stated above, ${\Psi }_{\downarrow }(x,{k}_{y})$ can also be calculated.

Nevertheless, this formalism also applicable for an infinite sample provided appropriate boundary conditions have been incorporated which are: $\psi (x=0,{k}_{y})=0$ and $\psi (x=-\infty ,{k}_{y})=0$.

### Calculation of charge conductance through edge states

The charge conductance $G$ through the edge states gets modified as an effect of gap opening in dispersion profile. Following the Landauer-Büttiker formula^54,68^, the charge conductance was calculated as

14 $$G=\frac{2{e}^{2}}{h}\left[1+\frac{1}{1+{e}^{[({E}_{gE}/2)-{E}_{F}]/{k}_{B}T}}-\frac{1}{1+{e}^{[-({E}_{gE}/2)-{E}_{F}]/{k}_{B}T}}\right]$$

where, ${E}_{F}$ is the Fermi energy (set to zero), ${k}_{B}$ is Boltzmann’s constant and $T$ is temperature. To be specific, all the conductance profiles in Fig. 5 were calculated for $T=300$ K and it was assumed that no disorder is present in the sample. Thus the conductance appears to be a linear function of ${E}_{gE}$, which will be highly nonlinear at low temperatures. It clearly indicates that when the spectrum is gapless, i.e. ${E}_{gE}=0$, then $G$ attains its maximum value of $2{e}^{2}/h$ and it reduces with increasing ${E}_{gE}$. Nevertheless, the conductance profile becomes quantized at absolute zero.

## Supplementary information

Supplementary information.

Supplementary information2.

## Data Availability

The authors declare that the main data supporting the findings of this study are available within the article and its supplementary information documents. Other relevant data are available from the corresponding author upon request.

## Code availability

MATLAB code for simulating dispersion spectra is shared in Supplementary Data.
