# Supplementary material for: Tuneable quantum spin Hall states in confined 1T' transition metal dichalcogenides
Source: Sci Rep. 2020 Apr 21;10:6670. doi: 10.1038/s41598-020-63450-5 (PMC7174349; doi:10.1038/s41598-020-63450-5)
Supplement: Supplementary file 1 — Supplementary information. [file 41598_2020_63450_MOESM1_ESM.pdf]

Supplementary Information

for

Tuneable quantum spin Hall states in confined 1*T′* transition metal dichalcogenides

Biswapriyo Das, Diptiman Sen and Santanu Mahapatra

Supplementary Fig. 1: Bulk band structures of $MoSe_2$

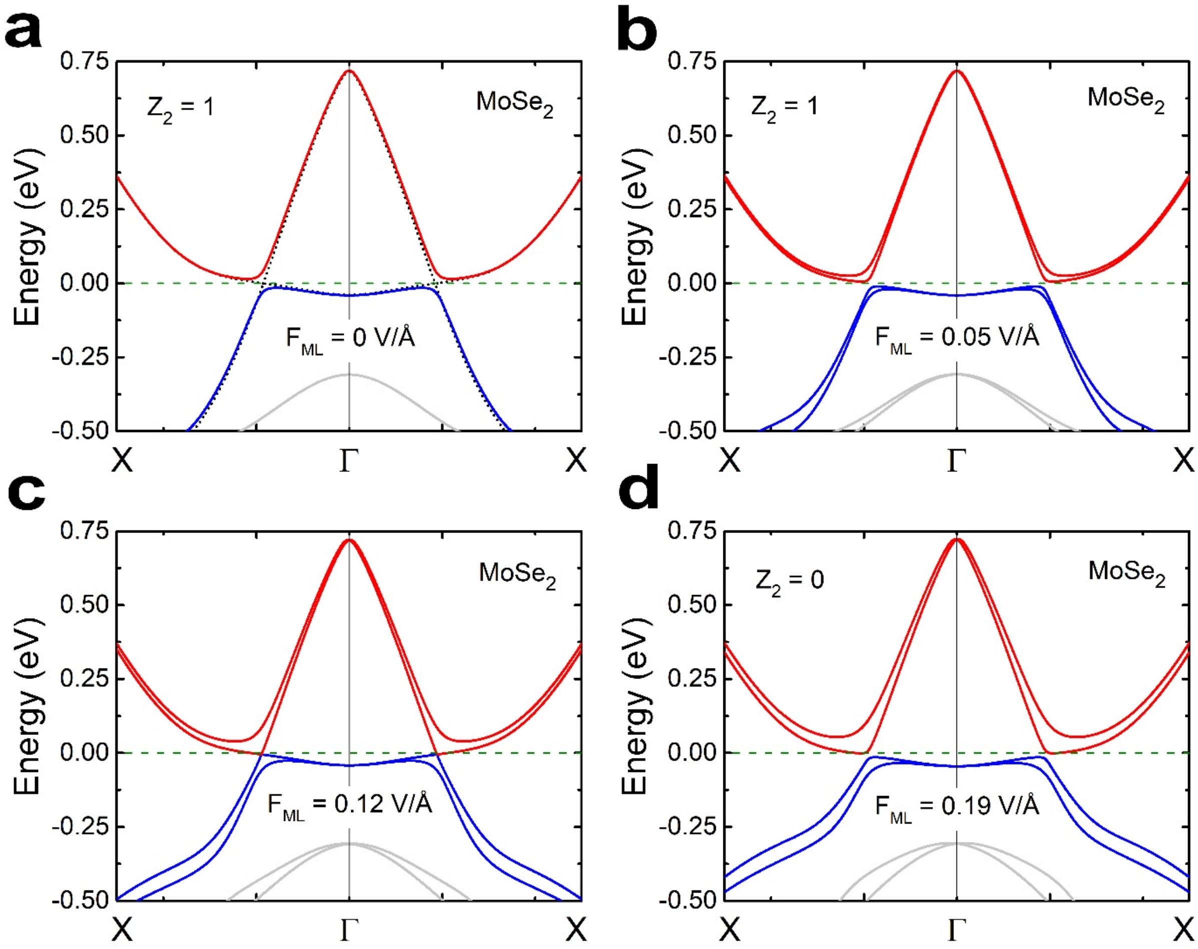

Supplementary Fig. 1: (a) depicts bulk band structure of $MoSe_2$ in absence of external electric field. The black dotted lines represent the band structure calculated without spin-orbit coupling. (b) – (d) represents the same for the monolayer field values of 0.05, 0.12 and 0.19 V/Å respectively. (c) represents that at 0.12 V/Å the dispersion becomes gapless.

Supplementary Fig. 2: Bulk band structures of WS_2

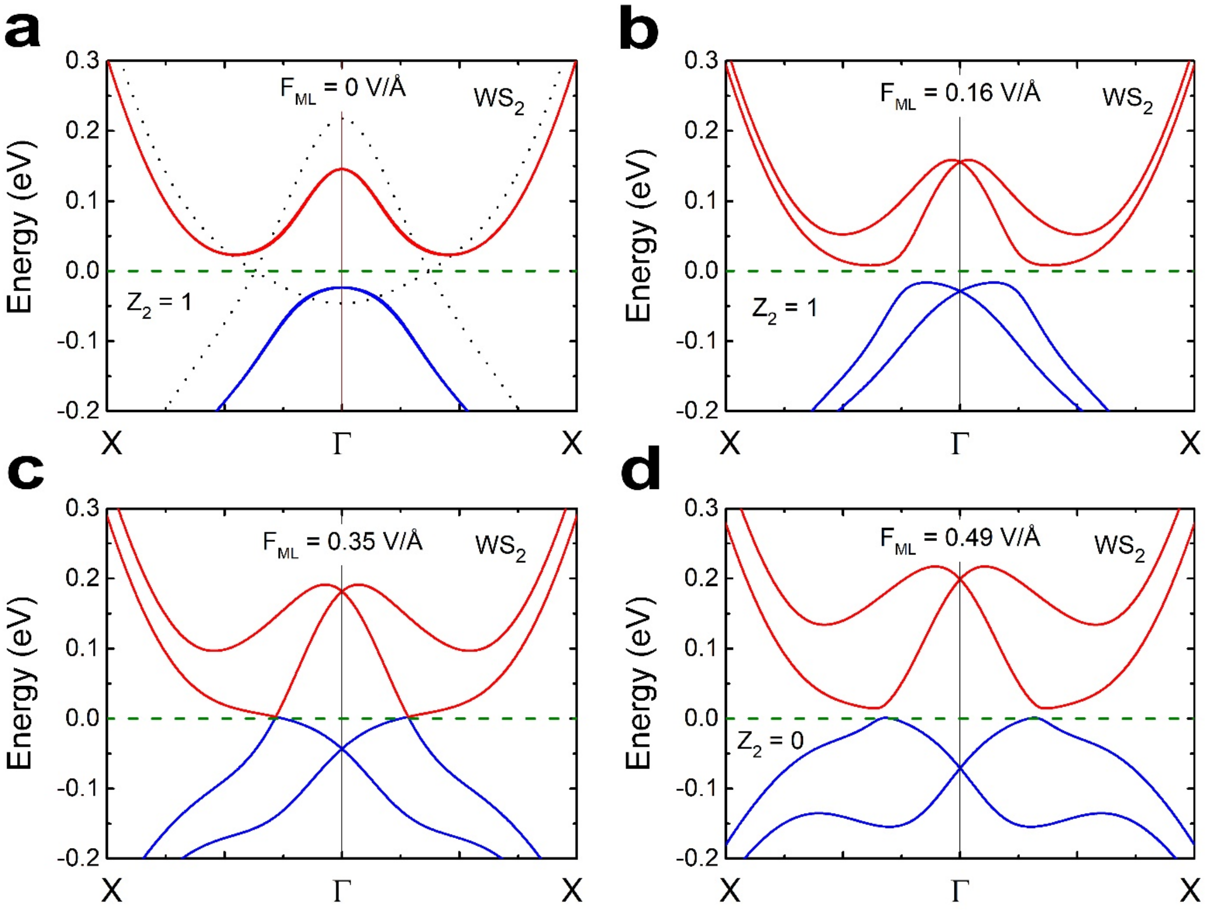

Supplementary Fig. 2: (a) depicts bulk band structure of WS_2 in absence of external electric field. The black dotted lines represent the band structure calculated without spin-orbit coupling. (b) – (d) represents the same for the monolayer field values of 0.16, 0.35 and 0.49 V/Å respectively. (c) represents that at 0.35 V/Å the dispersion becomes gapless.

Supplementary Fig. 3: Edge state spectra for $MoSe_2$

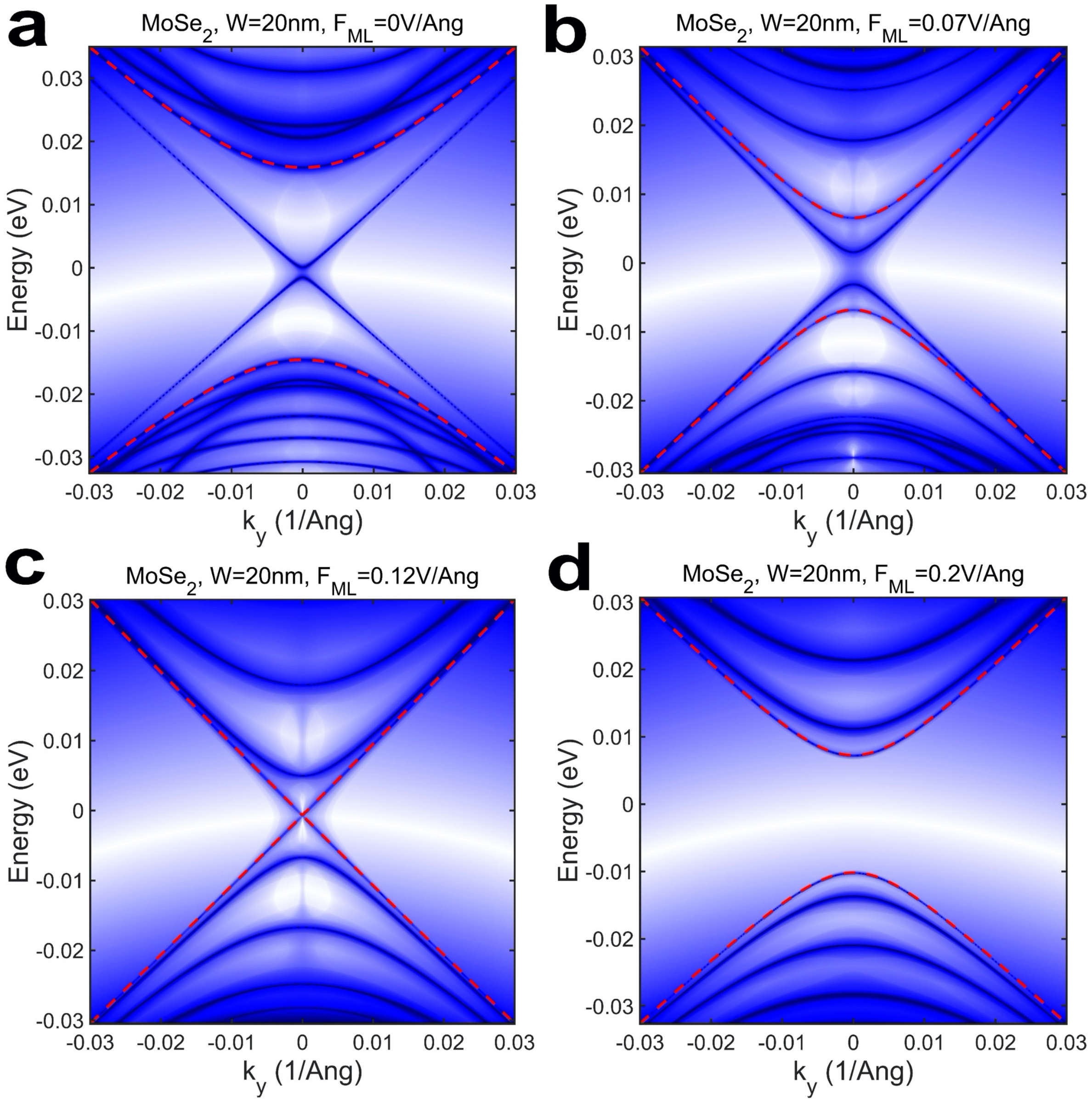

Supplementary Fig. 3: (a) – (d) represents the edge state dispersions for a 20nm wide $MoSe_2$ ribbon under the monolayer electric fields of 0, 0.07, 0.12 and 0.2 V/Å respectively. The red dashed lines denote the bulk bands. The critical electric field for topological phase transition was found to be 0.12 V/Å.

Supplementary Fig. 4: Edge state spectra for WS_2

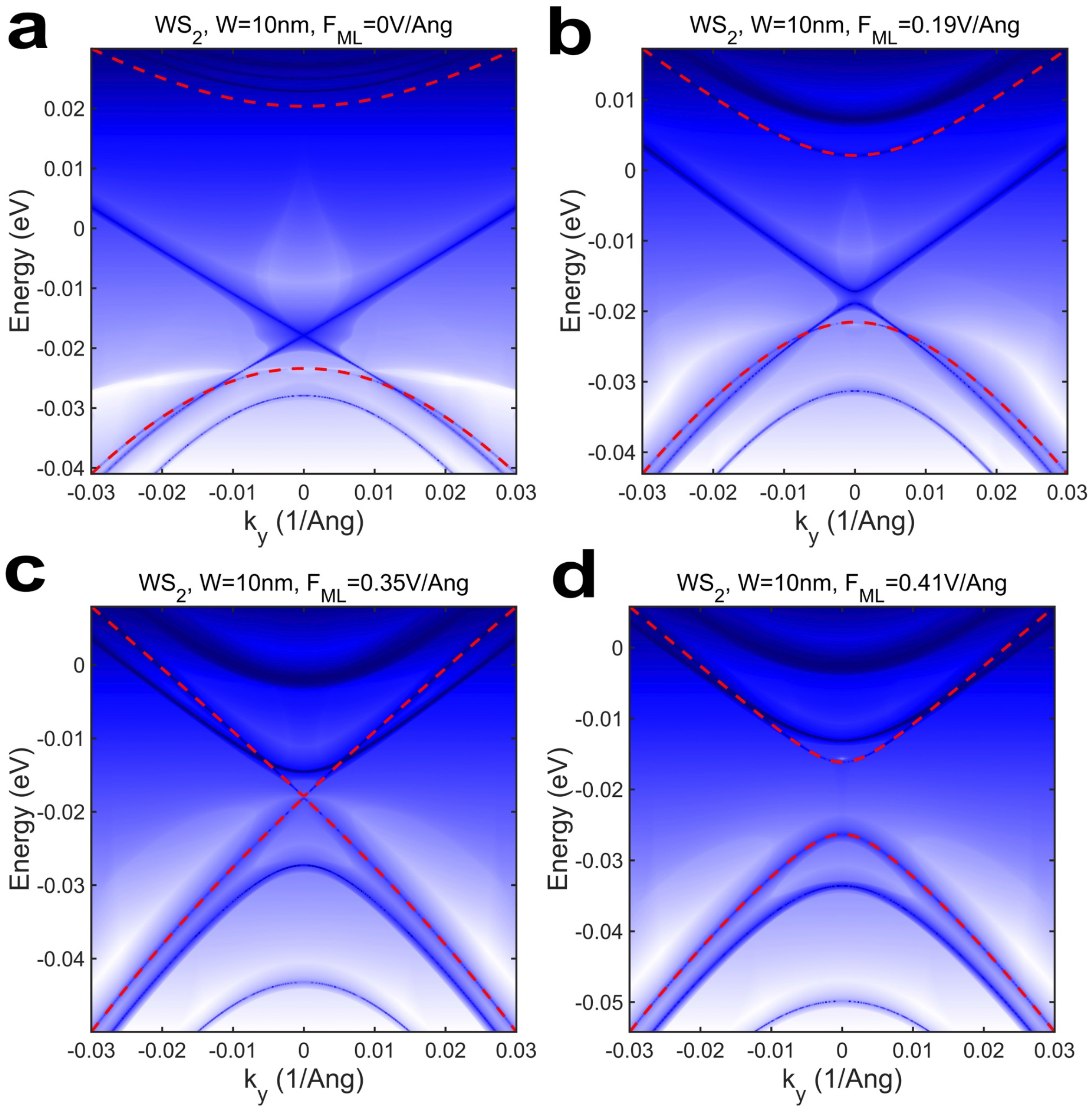

Supplementary Fig. 4: (a) – (d) represents the edge state dispersions for a 10nm wide WS_2 ribbon under the monolayer electric fields of 0, 0.19, 0.35 and 0.41 V/Å respectively. The red dashed lines denote the bulk bands. The critical electric field for topological phase transition was found to be 0.35 V/Å.

Supplementary Fig. 5: HSE Band structures

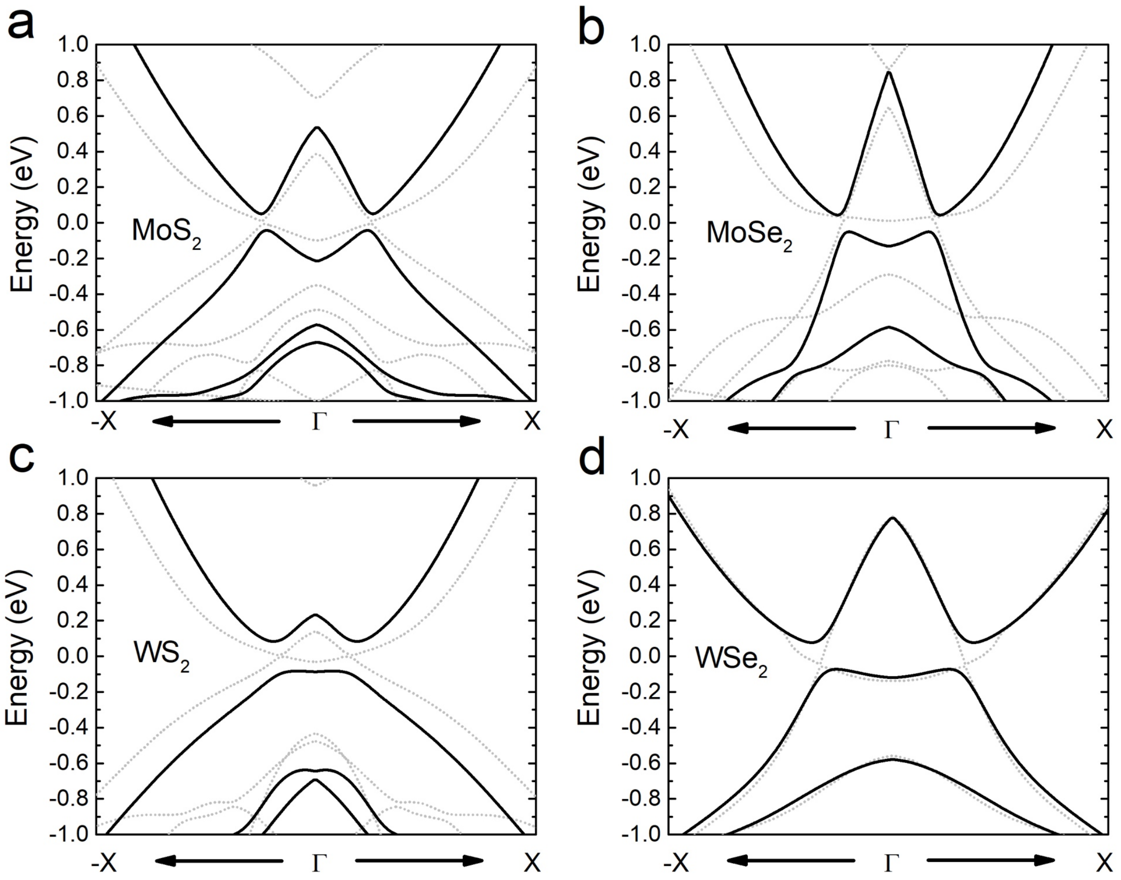

Supplementary Fig. 5: (a) – (d) represent the bulk band structures of 1T' MoS_2, $MoSe_2$, WS_2 and WSe_2 respectively in absence of electric field as computed using HSE functional. Here grey dotted lines represent the dispersions without considering spin-orbit coupling (SOC) and solid black lines indicate the dispersions with SOC. The role of SOC is to open a gap near the otherwise gapless Dirac cones. DFT calculations using HSE hybrid functional were carried out in VASP with PAW method with a plane-wave cutoff energy of 450 eV. For Brillouin zone sampling, a Γ-centered $6\times3\times1$ k-mesh was selected for all the materials. Electronic convergence was achieved when difference in energy of successive electronic steps became less than 10^{-4} eV. The fundamental band gap (E_g) of MoS_2, $MoSe_2$, WS_2 and WSe_2 were obtained to be 0.08 eV, 0.087 eV, 0.164 eV and 0.144 eV respectively. The inverted band gap

parameter δ_p was obtained as -0.543 eV, -0.86 eV, -0.237 eV and -0.783 eV and the parameter δ_d was calculated to be -0.216 eV, -0.132 eV, -0.086 eV and -0.120 eV respectively for MoS_2, $MoSe_2$, WS_2 and WSe_2.
