# Supplementary material for: Tuneable quantum spin Hall states in confined 1T' transition metal dichalcogenides
Source: Sci Rep. 2020 Apr 21;10:6670. doi: 10.1038/s41598-020-63450-5 (PMC7174349; doi:10.1038/s41598-020-63450-5)
Supplement: Supplementary file 2 — Supplementary information2. [file 41598_2020_63450_MOESM2_ESM.pdf]

MATLAB code developed to obtain the edge-state spectra of the four 1T' TMD materials:

```
% CODE FOR EDGE-STATE DISPERSIONS OF MoS2, MoSe2, WS2 & WSe2
% DEVELOPED @ NANO-SCALE DEVICE RESEARCH LABORATORY,
% DEPARTMENT OF ELECTRONIC SYSTEMS ENGINEERING (DESE)
% INDIAN INSTITUTE OF SCIENCE (IISc), BANGALORE;
% by,
% BISWAPRIYO DAS
%==================================================
% Kindly cite the original article entitled "Tuneable quantum spin Hall
% states in confined 1T' transition metal dichalcogenides" whenever use this code
%==================================================
clear all
close all
clc
format long
%% User inputs...
disp('Select the material...')
fprintf('\t Press 1 for MoS2\n\t Press 2 for MoSe2\n\t Press 3 for WS2\n\t Press 4 for WSe2\n\n')
material = input('');

disp('Select the Width (W) of the sample material...')
fprintf('\t Press 1 for 10nm\n\t Press 2 for 20nm\n\t Press 3 for 30nm\n\t Press 4 for 100nm\n\n')
W_select = input('');

disp('Select the magnitide of Monolayer Electric Field (FML) for the sample material...')
switch material
```

```matlab
    case 1
        fprintf('\t MoS2:  [(1-6) < (7=-Fc) < (8-14) < (15=0eV/Ang) < (16-22) < (23=Fc) < (24-29)]\n')
        fprintf('\t Choose a number between 1 to 29\n')
    case 2
        fprintf('\t MoSe2: [(1-5) < (6=-Fc) < (7-14) < (15=0eV/Ang) <(16-23) < (24=Fc) < (25-29)]\n')
        fprintf('\t Choose a number between 1 to 29\n')
    case 3
        fprintf('\t WS2:   [(1-4) < (5=-Fc) < (6-22) < (23=0eV/Ang) < (24-40) < (41=Fc) < (42-45)]\n')
        fprintf('\t Choose a number between 1 to 45\n')
    case 4
        fprintf('\t WSe2:  [(1=-Fc) < (2-11) < (12=0eV/Ang) < (13-22) < (23=Fc)]\n\n')
        fprintf('\t Choose a number between 1 to 23\n')
    otherwise
        disp('WRONG CHOICE')
end
FML_select = input('');

%% Define UNIVERSAL CONSTANTS
        q = 1.602e-19;              % electronic charge (C)
        h = 6.626e-34;              % Planck's constant (J-s)
        hbar = (6.626e-34)/(2*pi);  % modified Planck's constant (J-s)
        m0 = 9.11e-31;              % rest mass of electron (Kg)
        T = 300;                    % temperature (K)
        K = 1.38e-23;               % Boltzman constant (J/K)

%% Define MATERIAL SPECIFIC PARAMETERS
switch material
    case 1
```

```
    % MoS2
    material_char = 'MoS_2';
    deltap = (-0.41765)*q; %(J)
    deltad = (-0.13258)*q; %(J)
    mxp = (0.295)*m0; %(Kg)
    myp = (0.476)*m0; %(Kg)
    mxd = (0.920)*m0; %(Kg)
    myd = (2.32)*m0; %(Kg)
    v1 = (0.23)*1e5; %(m/s)
    v2 = (3.383)*1e5; %(m/s)
    Eg = (0.047)*q; %(J)
    Eg_inv = (0.55)*q; %(J)
    alpha = (0.159297)*q*1e-10; %(m)
    % FML_var for MoS2... %(V/m) [29 entries from DFT]
    FML_var = [-0.29713 -0.27598    -0.25471    -0.23503    -0.21204    -0.19168    -0.17116    -0.14835...
        -0.12794    -0.10681    -0.08291    -0.06271    -0.04234    -0.02092    0.0 0.02065 0.0416  0.0622...
        0.08267 0.10671 0.12765 0.1482  0.17075 0.19109 0.21196 0.23431 0.25452 0.27576 0.29692]*1e10;
    coldivide = 20;
case 2
    % MoSe2
    material_char = 'MoSe_2';
    deltap = (-0.71912)*q; %(J)
    deltad = (-0.040819)*q; %(J)
    mxp = (0.17468)*m0; %(Kg)
    myp = (0.28103)*m0; %(Kg)
    mxd = (3.1368)*m0; %(Kg)
    myd = (2.65)*m0; %(Kg)
    v1 = (0.285)*1e5; %(m/s)
```

```
    v2 = (3.421)*1e5; %(m/s)
    Eg = (0.0304)*q; %(J)
    Eg_inv = (0.76)*q; %(J)
    alpha = (0.27166)*q*1e-10; %(m)
    % FML_var for MoSe2... %(V/m) [29 entries from DFT]
    FML_var = [-0.19718 -0.18268    -0.16914    -0.15309    -0.13884    -0.12675    -0.11251    -0.09903...
        -0.08489    -0.07077    -0.05778    -0.04393    -0.02818    -0.01492    0.0 0.0145  0.02932 0.04221...
        0.05634 0.07055 0.08469 0.09885 0.11282 0.12545 0.13932 0.15306 0.16927 0.18368 0.19685]*1e10;
    coldivide = 20;
case 3
    % WS2
    material_char = 'WS_2';
    deltap = (-0.14533)*q; %(J)
    deltad = (-0.023416)*q; %(J)
    mxp = (0.27796)*m0; %(Kg)
    myp = (0.52731)*m0; %(Kg)
    mxd = (8.2)*m0; %(Kg)
    myd = (3.2)*m0; %(Kg)
    v1 = (0.845)*1e5; %(m/s)
    v2 = (2.931)*1e5; %(m/s)
    Eg = (0.0461)*q; %(J)
    Eg_inv = (0.169)*q; %(J)
    alpha = (0.173)*q*1e-10; %(m)
    % FML_var for WS2... %(V/m) [45 entries from DFT]
    FML_var = [-0.41465 -0.39399    -0.37592    -0.36361    -0.35466    -0.34309    -0.33271    -0.31188...
        -0.29126    -0.27023    -0.24863    -0.2288 -0.20791    -0.1877 -0.16648    -0.14559    -0.12455...
        -0.10374    -0.08295    -0.0629 -0.04178    -0.02082    0.0 0.02204 0.04307 0.06214 0.0829  0.1037...
        0.12457 0.14544 0.1664  0.18751 0.20784 0.22826 0.24891 0.27119 0.29137 0.31197 0.33277...
```

```
        0.3432  0.35336 0.36289 0.37408 0.39439 0.41494]*1e10;
      coldivide = 5;
   case 4
      % WSe2
      material_char = 'WSe_2';
      deltap = (-0.67832)*q; %(J)
      deltad = (-0.011813)*q; %(J)
      mxp = (0.16514)*m0; %(Kg)
      myp = (0.3611)*m0; %(Kg)
      mxd = (8.4)*m0; %(Kg)
      myd = (3.28)*m0; %(Kg)
      v1 = (0.38)*1e5; %(m/s)
      v2 = (3.542)*1e5; %(m/s)
      Eg = (0.023)*q; %(J)
      Eg_inv = (0.69)*q; %(J)
      alpha = (0.241375)*q*1e-10; %(m)
      % FML_var for WSe2... %(V/m) [23 entries from DFT]
      FML_var = [-0.17757 -0.16155    -0.14539    -0.12926    -0.11312    -0.0972 -0.08233    -0.06302...
         -0.04667    -0.03229    -0.01611    0.0 0.01615 0.03212 0.04657 0.06289 0.08186 0.09736...
         0.1132  0.1292  0.14532 0.16146 0.1775]*1e10;
      coldivide = 5;
   otherwise
      disp('WRONG CHOICE')
end

%% DEVICE PARAMETERS
W_var = [10, 20, 30, 100]*(1e-9); %(m)
W = W_var(W_select); %(m)
```

```
FML = FML_var(FML_select);
fprintf('\t FML = %f V/m\n',FML)

%% ~~~~~~~~~~~~~~~~~~~~~~BAND STRUCTURE CALCULATION~~~~~~~~~~~~~~~
ky_var = linspace(-0.03*1e10, 0.03*1e10, 1001);
ky0pt = find(ky_var == 0);
kx = linspace(-0.3*1e10, 0.3*1e10, 1001);
kx0pt = find(kx == 0);
for j = 1:1:length(ky_var)
    ky = ky_var(j);
    %% Calculation of BULK BAND STRUCTURE
    Ep = -deltap - (((hbar^2).*(ky.^2))./(2*myp)) - (((hbar^2).*(kx.^2))./(2*mxp));
    Ed = deltad + (((hbar^2).*(ky.^2))./(2*myd)) + (((hbar^2).*(kx.^2))./(2*mxd));
    X = v1*hbar.*kx;
    Y = sqrt(-1)*v2*hbar.*ky;

    Energy1 = ((Ep+Ed)./2) + sqrt( ((Ep-Ed)./2).^2 + (X+alpha.*FML).^2 - Y.^2 );
    Energy2 = ((Ep+Ed)./2) + sqrt( ((Ep-Ed)./2).^2 + (X-alpha.*FML).^2 - Y.^2 );
    Energy3 = ((Ep+Ed)./2) - sqrt( ((Ep-Ed)./2).^2 + (X+alpha.*FML).^2 - Y.^2 );
    Energy4 = ((Ep+Ed)./2) - sqrt( ((Ep-Ed)./2).^2 + (X-alpha.*FML).^2 - Y.^2 );

    E3D1(j,:) = ((Ep+Ed)./2) + sqrt( ((Ep-Ed)./2).^2 + (X+alpha.*FML).^2 - Y.^2 );
    E3D2(j,:) = ((Ep+Ed)./2) + sqrt( ((Ep-Ed)./2).^2 + (X-alpha.*FML).^2 - Y.^2 );
    E3D3(j,:) = ((Ep+Ed)./2) - sqrt( ((Ep-Ed)./2).^2 + (X+alpha.*FML).^2 - Y.^2 );
    E3D4(j,:) = ((Ep+Ed)./2) - sqrt( ((Ep-Ed)./2).^2 + (X-alpha.*FML).^2 - Y.^2 );

    CBmin(j) = min(Energy1/q); % Energy of Conduction Band minima
    VBmax(j) = max(Energy3/q); % Energy of Valence Band maxima
    mid(j) = ((CBmin(j) + VBmax(j))/2);
```

```
%% Calculation of EDGE-STATE DISPERSION
bound1 = VBmax(1); %eV % Lower bound for energy dispersion
bound2 = CBmin(1); %eV % Upper bound for energy dispersion

E_test = (linspace(bound1,bound2,1000))*q; %J % No. of sample points between bounds is 1000
E_mat(j,:) = E_test;

for k = 1:1:length(E_test)
    E = E_test(k);
    % Computation of parameters
    A = -deltap - (((hbar^2).*(ky.^2))./(2*myp)) - E;
    B = (hbar^2)/(2*mxp);
    C = deltad + (((hbar^2).*(ky.^2))./(2*myd)) - E;
    D = (hbar^2)/(2*mxd);
    P = sqrt(-1)*v2*hbar.*ky;
    Q = sqrt(-1)*v1*hbar;
    % Calculation of lambda
    coeff4 = B.*D;
    coeff3 = 0;
    coeff2 = Q.^2 + A.*D - B.*C;
    coeff1 = -2.*Q.*alpha.*FML;
    coeff0 = -( A.*C + P.^2 - (alpha.*FML).^2 );
    % Polynomial construction
    poly1 = [coeff4 coeff3 coeff2 coeff1 coeff0];
    % Roots of polynomials
    lamset1 = roots(poly1);
    lam1 = (lamset1(1));
    lam2 = (lamset1(2));
```

```
        lam3 = (lamset1(3));
        lam4 = (lamset1(4));
        % Calculation of Eigenfunctions
        psid1 = (A+B*(lam1^2))/(P-alpha*FML+Q*lam1);
        psid2 = (A+B*(lam2^2))/(P-alpha*FML+Q*lam2);
        psid3 = (A+B*(lam3^2))/(P-alpha*FML+Q*lam3);
        psid4 = (A+B*(lam4^2))/(P-alpha*FML+Q*lam4);

        fun = 4 * sinh((W/2)*(lam1-lam2)) * sinh((W/2)*(lam3-lam4)) * (psid1*psid2 + psid3*psid4) -...
            4 * sinh((W/2)*(lam1-lam3)) * sinh((W/2)*(lam2-lam4)) * (psid1*psid3 + psid2*psid4) +...
            4 * sinh((W/2)*(lam2-lam3)) * sinh((W/2)*(lam1-lam4)) * (psid2*psid3 + psid1*psid4);

        funval(j,k) = abs(fun);
    end
    fprintf('ky point complete for VB: %d out of %d\n',j,length(ky_var))
end

%% Extract Energy Eigenvalues
clear Esol_temp Esol color1 color2 color3 mycolor
z = length(E_test);
for j = 1:1:length(ky_var)
    E_test = E_mat(j,:);
    funvaldata = funval(j,:);
    funvaldata_sort = sort(funvaldata,'ascend');
    funvaldata_select = funvaldata_sort(1:z);
    for k = 1:1:length(funvaldata_select)
        Esol_temp(k) = (E_test(find(funvaldata==funvaldata_select(k))))/q;
    end
    Esol(j,:) = (Esol_temp(:));
```

```
end
Esol = transpose(Esol);

    %% Define Color Map for plotting the Edge-State Dispersions
    clear color1 color2 color3 mycolor colorlength cmap
    colorlength = length(E_test);
        color1 = [zeros(1,floor(colorlength/coldivide)),linspace(0,1,colorlength-floor(colorlength/coldivide))];
        color2 = [zeros(1,floor(colorlength/coldivide)),linspace(0,1,colorlength-floor(colorlength/coldivide))];
        color3 = [linspace(0.5,1,floor(colorlength/coldivide)),ones(1,colorlength-floor(colorlength/coldivide))];
    mycolor(1,:) = color1;
    mycolor(2,:) = color2;
    mycolor(3,:) = color3;
    mycolor = (transpose(mycolor));

    %% Generate filename and figure title
    format short
    FMLforfig = FML/1e10;
    filename = strcat(char(material_char),'_W',num2str(W/1e-9),'_FML',num2str(FML_select),'.mat');
    fig_title = strcat(char(material_char),', W=',num2str(W/1e-9),'nm, F_M_L= ',char(' '),char(num2str(FMLforfig)),'V/Ang');
    %% Plotting Edge State dispersions
        figure(1)
        for k = z:-1:1
            plot(ky_var*1e-10,Esol(k,:),'.','MarkerEdgeColor',mycolor(k,:))
%               plot(ky_var*1e-10,Esol(k,:),'s','MarkerFaceColor',mycolor(k,:),'MarkerEdgeColor',mycolor(k,:))
            hold on
        end
        plot(ky_var*1e-10,VBmax,'r--','LineWidth',3)
        hold on
```

```
plot(ky_var*1e-10,CBmin,'r--','LineWidth',3)
hold on
title(fig_title,'FontWeight','normal','FontSize',28)
xlim([-0.03 0.03])
ylim([-0.05 0.05])
set(gca, 'Layer', 'top')
set(gca,'linewidth',2)
xxx = get(gca,'XTickLabel');
set(gca,'XTickLabel',xxx,'fontsize',22,'FontWeight','normal')
set(gca,'XTickLabelMode','auto')
xlabel('k_y (1/Ang)','FontSize',28)
ylabel('Energy (eV)','FontSize',28)
pbaspect([1 1 1])
xticks([-0.03:0.01:0.03])
yticks([-0.05:0.01:0.05])
```
